# Network Formation in Poly(diallyldimethylammonium chloride)/Polyphosphoric Acid Polyelectrolyte Complexes and Their Role in Sand Stabilization

**DOI:** 10.1021/acs.langmuir.6c02727

**Published:** 2026-08-31

**Authors:** Rui Zhi Liu, Kong Teng Ling, JitKang Lim

**Affiliations:** † School of Chemical Engineering, Universiti Sains Malaysia, Nibong Tebal, Penang 14300, Malaysia; ‡ Schlumberger (M) Sdn Bhd, Kuala Lumpur 50450, Malaysia

## Abstract

Soil erosion remains a pressing global environmental challenge, and polyelectrolyte complexes (PECs) have emerged as promising stabilizing agents for soil erosion control. However, the mechanistic basis of their performance, particularly the complex interplay between the structure of PECs, rheological behavior, and the associated flow field within the surrounding environment, remains poorly understood. In this study, we constructed PECs by combining a short-chain polyanion and a long-chain polycation to form a polymeric network, using polyphosphoric acid (PPA) and poly­(diallyldimethylammonium chloride) (PDDA) as the model components. Electrophoretic mobility (EPM) measurements, hydrodynamic diameter analysis by dynamic light scattering (DLS), and quartz crystal microbalance with dissipation (QCM-D) collectively reveal an electrostatically driven self-assembly process in which long-chain PDDA provides the scaffold while short-chain PPA acts as a molecular connector. Rheological measurements show that the shear resistance of the PDDA/PPA network, characterized by the effective shear rate γ̇ and the characteristic shear stress τ at the *G*′ maximum, can be tuned by adjusting the PPA dosage. Macroscale hydrodynamic shear tests further demonstrate a strong positive correlation between consolidated-sand stability and the rheology-derived network strength, with both γ̇ and τ at the *G*′ maximum reaching a maximum at a PDDA:PPA monomer molar ratio of 1:5. Scanning electron microscopy coupled with energy-dispersive X-ray spectroscopy (SEM-EDX) directly visualizes interparticle physical bridging that promotes sand aggregation. Collectively, these results suggest that the PPA-regulated strength of the PECs network is an important factor influencing sand-stabilization performance. Such findings shift the design philosophy of sand stabilizers from conventional surface adhesion toward the internal structural engineering of particle-bridging networks.

## Graphical abstract

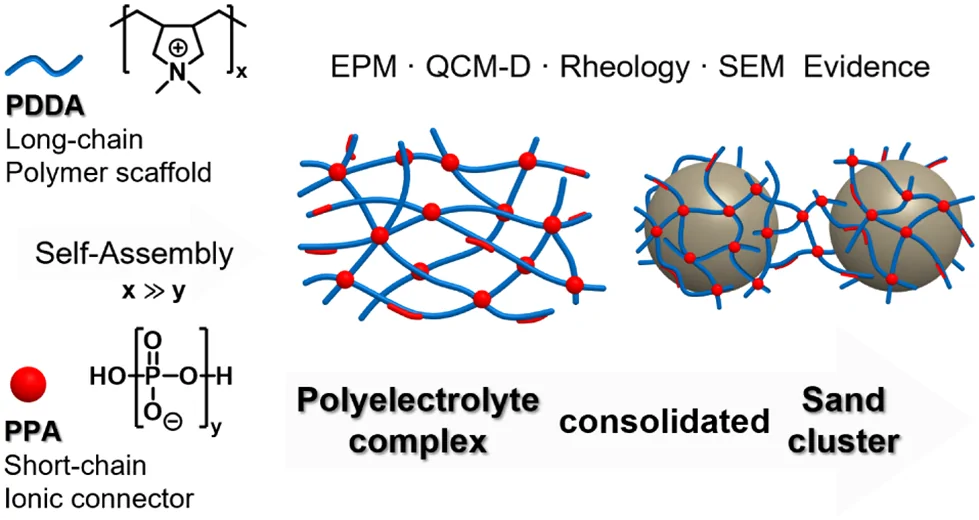

## 1 Introduction

Soil loss can lead to agricultural yield reduction, infrastructure damage, and ecological imbalance.^1,2^ Polyelectrolyte complexes (PECs) exhibit tunable charge characteristics and spontaneous self-assembly behavior^3^ and have also attracted attention as promising sand-stabilizing materials.^4,5^ Although numerous studies have demonstrated that PECs can enhance crust strength and resistance to water erosion, most existing research still focuses primarily on evaluating macroscopic performance,^6–8^ with a relatively limited mechanistic understanding of how PECs stabilize sand.

Although a complete mechanistic understanding of PEC systems in soil stabilization remains incomplete, several experimental observations and mechanistically motivated hypotheses have been reported. For instance, some studies have proposed that hydrophobic interactions play a dominant role in sand stabilization.^9^ In this view, PECs are considered as block copolymer-like structures, where uncompensated charged segments form hydrophilic blocks and charge-neutralized segments form hydrophobic blocks.^10^ The hydrophilic blocks are adsorbed onto sand surfaces via electrostatic interactions, whereas the hydrophobic blocks further stabilize the attachment through hydrophobic interactions and compress the sand aggregates into a more compact structure.^11^ However, such interpretations often overlook the potential contribution of the internal network structure of PECs to their macroscopic sand-stabilization performance.

Other studies have shown that, as the fraction of anionic polyelectrolyte increases, PEC systems can undergo a transition from a low-viscosity solution to a gel-like state, accompanied by pronounced increases in zero-shear viscosity, storage modulus, and relaxation time.^12^ These changes were attributed to the presence of enhanced physical cross-linking and the formation of a network-like structure within PECs;^12,13^ however, direct evidence of the formation of such a network between the sand-PEC-sand system, or more rigorous indirect verification, is still lacking. Building on the assumption of polymeric network formation capability, recent studies have reported that increasing the proportion of anionic polyelectrolyte correlates with enhanced mechanical strength and improved resistance to water-induced erosion in treated sand.^14^ On this basis, we hypothesized that sand-PEC cluster stability is governed by the strength of the underlying polymeric network.

Overall, these mechanistic hypotheses are all logically reasonable but remain at the level of speculation or preliminary validation, lacking systematic and multiscale experimental evidence and without the support of a theoretical framework. Consequently, a substantial knowledge gap persists between the sand-stabilization performance of PECs and their underlying microscopic mechanisms, which in turn hampers further advances in material optimization and molecular design.

To elucidate the sand-stabilization mechanism of PECs, we selected a PDDA/PPA pair as an asymmetric model system because the combination of a long-chain cationic component and a short-chain anionic component allows the respective functions of scaffold formation and ionic connection to be more clearly distinguished during polymeric network formation. Specifically, long-chain poly­(diallyldimethylammonium chloride) (PDDA) with an average degree of polymerization of approximately 2787 (Figure 1a, left) mainly serves as a permeable polymer scaffold, whereas short-chain, multivalent, negatively charged polyphosphoric acid (PPA) with an average degree of polymerization of approximately 6 ^15^ (Figure 1a, right) can penetrate the inter- and intrachain voids of PDDA and act primarily as a “molecular connector” through multipoint ionic bridging, thereby constructing a tunable polymeric network (Figure 1b). This asymmetric design allows variations in PPA content to more directly regulate the density of ionic connectors and thus the overall strength of the PEC network, thereby making the influence of network strength on sand-cluster stability easier to interpret. On this basis, we hypothesize that the resulting network can cover particle surfaces and form interconnected bridges across particles, thereby promoting sand aggregation and reinforcement (Figure 1c).

**Figure 1:**
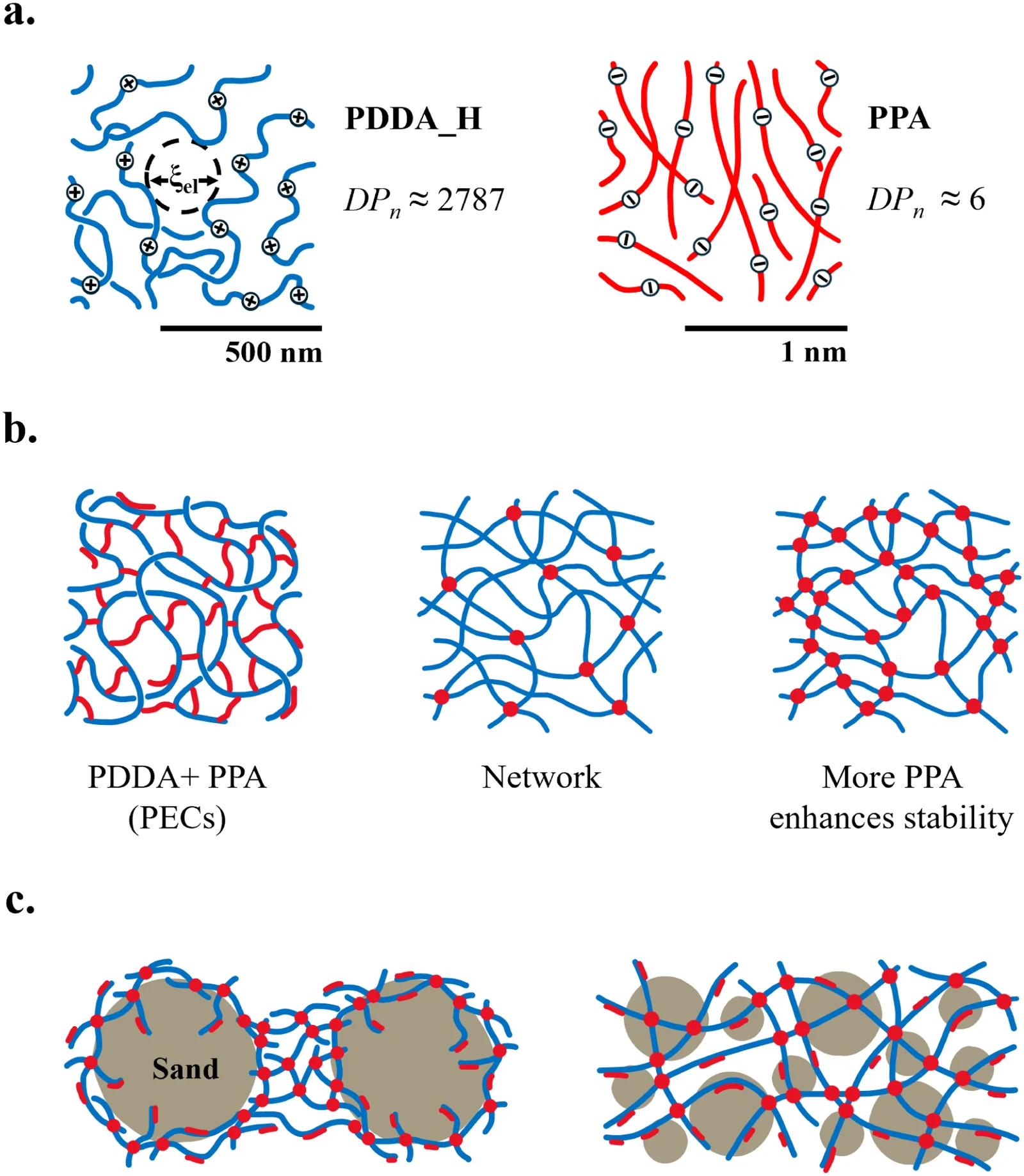
Schematic of network formation in PDDA/PPA polyelectrolyte complexes (PECs) and their role in sand stabilization. (a) Molecular structural features of PDDA and PPA. (b) PDDA and PPA undergo electrostatic complexation to form PECs; through multisite ionic bridging by PPA, the system evolves into an interconnected polymeric network. Increasing the PPA content increases the number of anchoring points and enhances network stability. (c) The resulting network coats sand-grain surfaces and bridges across particles, promoting aggregation and strengthening of previously dispersed sand grains (not drawn to scale; the polymer network is enlarged to provide a pictorial illustration for the hypothesized sand-bridging mechanism).

To test this hypothesis, we planned our research activities as follows: (i) provide interfacial evidence of electrostatic self-assembly of the PEC network using electrophoretic mobility (EPM), hydrodynamic diameter measurements by dynamic light scattering (DLS), and quartz crystal microbalance with dissipation (QCM-D); (ii) use oscillatory rheology to recast frequency sweeps into two metrics, the shear rate (γ̇) and the characteristic shear stress (τ), which provide quantitative measures of how PECs respond to deformation across relevant length and time scales, thereby reflecting the presence and connectivity of a polymeric network rather than simple viscous flow.^16–20^ In the context of sand stabilization, these measurements directly link network integrity to the ability of PECs to transmit and withstand hydrodynamic stresses within granular media, offering mechanistic insight beyond macroscopic erosion resistance; (iii) perform rotational shearing tests, converting the critical rotational speed at sand-cluster disintegration into equivalent shear rate/stress to establish a structure–property relationship between PEC network strength and the mechanical stability of PECs-stabilized sand under hydrodynamic erosion; and (iv) provide direct visualization of polymeric network formation via SEM/EDX.

## 2 Materials and Methods

### 2.1 Materials

The quartz sand used in this study was a commercially available standard sand (Sigma-Aldrich, 50–70 mesh, main component SiO_2_) and was used as received without further treatment. The cationic polyelectrolyte was poly­(diallyldimethylammonium chloride) (PDDA), employed in two molecular-weight grades: PDDA_M (Sigma-Aldrich, 20 wt % aqueous solution; Mw = 2.0–3.5 × 10^5^ g·mol^–1^) and PDDA_H (high-molecular-weight grade; Mw = 4.0–5.0 × 10^5^ g·mol^–1^). The anionic polyelectrolyte was polyphosphoric acid (PPA; Sigma-Aldrich, ∼115%, based on H_3_PO_4_), which was used as received.

### 2.2 Preparation of PECs

In all formulations, the monomer-based molar amount of PDDA solid was fixed at 0.00625 mol (corresponding to 1.0 g). Different PDDA:PPA monomer ratios (1:1, 1:2, 1:3, 1:4, 1:5, 1:6, 1:7, and 1:8) were obtained by varying the amount of PPA added, with feed compositions calculated based on the monomer molar masses (PDDA, ∼161.1 g·mol^–1^; PPA, ∼80.0 g·mol^–1^). It should be emphasized that, throughout this work, all reported molar quantities are defined on a monomer-equivalent basis rather than per polymer chain; the rationale for this definition is provided in Supporting Information S1.

For each PEC preparation, a mass-based protocol was adopted. Specifically, 5.0 g of a 20 wt % aqueous PDDA solution (corresponding to 1.0 g of PDDA) was accurately weighed, which was selected to reduce relative weighing errors and ensure reliable and reproducible sample preparation. Subsequently, a calculated amount of PPA was added to the PDDA solution according to the specified PDDA:PPA monomer molar ratio (Table 1). The mixture was then magnetically stirred for 40 min to ensure a thorough mixing. The resulting PEC dispersions with different PDDA:PPA molar ratios were optically transparent, homogeneous single-phase systems and were used for subsequent characterization and performance tests.

**Table 1:** Formulations of PDDA/PPA Polyelectrolyte Complexes Prepared at Different Monomer-Equivalent Molar Ratios

PDDA:PPA monomer-equivalent molar ratio	PDDA solution feed amount (20 wt %, g)	PDDA solid content (g)	PDDA amount (monomer-equivalent mol)	PPA feed amount (g)	PPA amount (monomer-equivalent mol)
1:1	5	1	6.21 × 10^–3^	0.497	6.21 × 10^–3^
1:2	5	1	6.21 × 10^–3^	0.994	1.24 × 10^–2^
1:3	5	1	6.21 × 10^–3^	1.490	1.86 × 10^–2^
1:4	5	1	6.21 × 10^–3^	1.987	2.48 × 10^–2^
1:5	5	1	6.21 × 10^–3^	2.484	3.11 × 10^–2^
1:6	5	1	6.21 × 10^–3^	2.981	3.73 × 10^–2^
1:7	5	1	6.21 × 10^–3^	3.478	4.35 × 10^–2^
1:8	5	1	6.21 × 10^–3^	3.974	4.97 × 10^–2^

### 2.3 Electrophoretic Mobility and Hydrodynamic Size Measurements

The electrophoretic mobility (EPM) and hydrodynamic size of the samples were measured using a Zetasizer Nano ZS (Malvern Instruments, UK) at 25 °C. The samples tested were PEC dispersions prepared by mixing either PDDA_M or PDDA_H with PPA at different molar ratios. All samples were diluted with deionized water (electrical resistivity ≈ 18 MΩ·cm) or NaCl solutions (0.01, 0.1, 1, 10, and 100 mM) to a final PDDA concentration of 20 ppm, without pH adjustment. For comparison, the EPM values of the individual PDDA_M, PDDA_H, and PPA solutions were also measured. All measurements were performed in triplicate, and the average values are reported. For a selected sample (PDDA_H:PPA = 1:2), the hydrodynamic size, polydispersity index (PDI), and mean scattering intensity were further monitored continuously for 120 min in deionized water and 100 mM NaCl to evaluate the time-dependent stability of the dispersion.

### 2.4 QCM-D (Quartz Crystal Microbalance with Dissipation) Analysis

QCM-D was performed on a Q-Sense E1 (Biolin Scientific, Sweden) with SiO_2_-coated quartz sensors at 25 ± 0.1 °C and a flow rate of 0.34 μL min^–1^. After establishing a baseline in deionized water, PDDA_H solution (2000 ppm, 5 min) and PPA solution (500 ppm, 5 min) were alternately injected for five cycles, followed by a final rinse with deionized water to remove unbound species, yielding a PDDA/PPA composite layer. The frequency shift (Δ*f*) and dissipation (Δ*D*) were recorded in real time and analyzed at the third overtone (*n* = 3); Δ*f* qualitatively indicates mass adsorption, whereas Δ*D* reflects viscoelasticity.

According to the Sauerbrey relation, for a smooth and rigidly adsorbed film, the change in resonant frequency is proportional to the added mass, as expressed below.

1 $$\Delta m=-\frac{P_{q}t_{q}}{f_{0}}\cdot \frac{\Delta f}{n}=-C_{\mathrm{QCM}‐D}\frac{\Delta f}{n}$$

In this expression, *f*_0_ is the resonance frequency of the AT-cut quartz crystal, *P*_q_ is the density of quartz, *t*_q_ is the crystal thickness, *C*_QCM‑D_ is the mass-sensitivity constant (17.7 ng cm^–2^ Hz^–1^), *n* is the overtone number, Δ*f* is the frequency shift, and Δ*m* is the mass change.

### 2.5 Rheometry Measurements

Rheological measurements were carried out on an Anton Paar MCR 102 rotational rheometer at 25 °C using a CC17 concentric-cylinder geometry (inner cylinder diameter, 16.662 mm; outer cylinder diameter, 18.083 mm; gap, 0.711 mm). PEC samples prepared from high-molecular-weight PDDA_H and medium-molecular-weight PDDA_M with various PDDA:PPA molar ratios were tested. For all measurements, preprepared PEC samples with different PDDA:PPA molar ratios were loaded into the rheometer. Because the compositions of these PECs varied with formulation, the total mass load was adjusted according to sample composition rather than kept constant. In all cases, the loading amount was calculated such that the monomer-equivalent amount of PDDA introduced into the rheometer was identical, namely 1.655 mmol. During sample loading, the PECs were gently introduced along the cup wall to minimize air entrapment. After loading, the sample was allowed to rest for 1 min and was then subjected to oscillatory angular frequency sweeps at a fixed strain of 50%, during which the storage modulus (*G*′) was recorded. All measurements were performed in triplicate.

### 2.6 Mechanical Stability Testing

For the mechanical stability tests, 1.0 g of quartz sand was mixed with an amount of PECs corresponding to 17,300 times the theoretical dosage required to form monolayer coverage on the sand surface (specific calculations are provided in Supporting Information S2), giving a PEC mass of approximately 0.20 g. The stabilization effect in this study does not rely solely on the adsorption of a single polymer layer on the sand surface but involves the formation of interparticle bridging networks by PECs between sand particles. Therefore, additional polymer is required in the actual system to achieve interparticle connections and three-dimensional network construction, and the required dosage is inevitably higher than the theoretical monolayer coverage. When mixing sand with PECs at different PDDA:PPA molar ratios, the molar amount of PDDA introduced into the sand system was kept constant at 0.20 mmol. The mixture was then molded into a spherical shape with a diameter of approximately 1 cm for mechanical stability testing. All measurements were performed in triplicate.

The resistance of the sand clusters to hydrodynamic erosion was evaluated by using a custom-assembled device. In brief, 1800 mL of deionized water was placed in a glass beaker, inside of which a mesh basket was fixed to hold the sand cluster, while an overhead stirrer was mounted vertically above the beaker to generate a circumferential water flow (Figure 2). The test was conducted under a linearly accelerated protocol: the rotational speed was increased from 0 to 660 rpm within 9 s. The entire process was recorded using a camera, and the rotational speed at which a through-crack formed in the sand cluster followed by rapid disintegration was defined as the critical rotational speed (*n*_c_). The corresponding critical shear rate and critical shear stress were then calculated from *n*_c_ using the following steps, as described by eqs 2–6.

**Figure 2:**
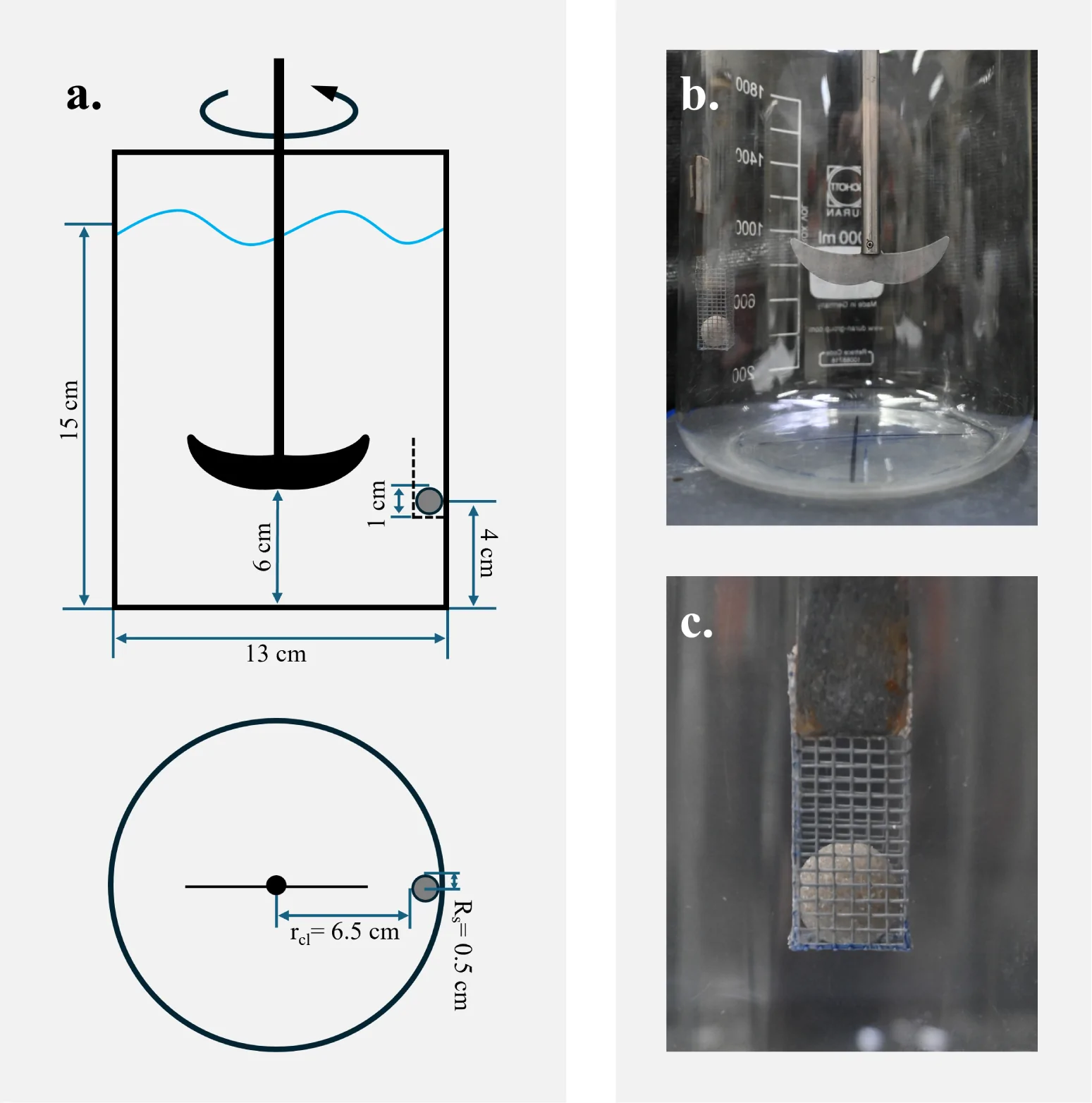
Schematic illustration of the water-shear erosion test setup for PEC-sand aggregates and the corresponding system parameters. (a) Schematic diagram of the experimental setup. (b) Photograph of the overall experimental apparatus. (c) Enlarged view of a PEC-sand aggregate placed inside the mesh basket.

Based on the force model of a spherical sand cluster in flowing water, the tangential hydrodynamic drag exerted on the sand-cluster surface can be expressed as^21^

2 $$F_{\mathrm{shear}}=C_{d}\tau_{w}\pi R_{s}^{2}$$

where *C*_d_ is the drag coefficient (the value and calculation procedure are provided in Supporting Information S3), τ_w_ = μγ̇ is the shear stress of the flowing water, and *R*_s_ is the radius of the sand cluster.

The sand cluster is held together by the internal cohesion provided by PECs, and its resistance to fracture can be expressed as^22^

3 $$F_{\mathrm{cohesive}}=\tau_{c}\pi R_{s}^{2}$$

where τ_c_ is the critical cohesive shear stress of the sand cluster.

When *F*_shear_ ≥ *F*_cohesive_, the sand cluster fails, yielding:

4 $$\tau_{c}=C_{d}\mu \mathrm{\gamma ̇}$$

Considering the rotational flow field, the shear rate can be approximated as^23^

5 $$\mathrm{\gamma ̇}=\frac{\omega_{c}r_{cl}}{R_{s}}$$

where $\omega_{c}=\frac{2\pi n_{c}}{60}$ is the angular velocity and *r*_cl_ is the distance between the stirrer (impeller) and the center of the sand cluster.

Finally, the shear stress at sand-cluster disintegration can be calculated using

6 $$\tau_{c}=C_{d}\mu \frac{\omega r_{cl}}{R_{s}}$$

By approximating the near-wall flow as a Couette-type flow and using the CFD-simulated velocities before and after entering the mesh basket, namely 30.1 and 18.3 cm/s, respectively, the ratio of shear force with and without the mesh basket can be estimated as proportional to the velocity ratio. This gives a value of approximately 0.61, indicating that the presence of the mesh basket reduces the effective shear force experienced by the sand clusters. Therefore, the current shear force analysis, which does not account for the presence of the mesh basket, likely overestimates the actual shear force within the basket. However, it should be noted that this estimation is based on a simplified planar approximation, and the local shear force ratio may vary substantially depending on the position within the flow field. Through these shear-related relationships, the experimentally measured critical rotational speed can be converted into an equivalent shear rate and shear stress, enabling a quantitative assessment of how PECs with different compositions contribute to the resistance of sand clusters against hydrodynamic shear-induced disintegration.

### 2.7 SEM and EDX Characterization

The surface morphology and elemental distributions of sand particles treated with PECs were examined using field-emission scanning electron microscopy (FESEM, Quanta FEG 450, FEI, USA) equipped with energy-dispersive X-ray spectroscopy (EDX). For sample preparation, 1.0 g of quartz sand was thoroughly mixed with 0.2 g of PECs, followed by drying at 60 °C under vacuum. The dried samples were directly subjected to SEM and EDX analyses. As a control, blank quartz sand without PEC treatment was prepared and characterized under identical conditions.

## 3 Results and Discussion

### 3.1 Electrostatically Bridged Formation of PDDA/PPA Networks

Previous studies on PECs have mostly focused on symmetric long-chain/long-chain polyelectrolyte combinations, such as PDDA/polystyrenesulfonate (PSS),^24^ PDDA/potassium humate (PH),^6,7^ PDDA/poly­(acrylic acid) (PAA),^8^ and others.^10^

In contrast, this work explores the design of PECs for soil consolidation by using an asymmetrical, bimodal system composed of the long-chain polycation PDDA and the short-chain polyanion PPA. The selection of the PDDA/PPA pair was further supported by the control experiments presented in Supporting Information S4, in which other PEC systems, such as PDDA/PSS and PDDA/PAA, were evaluated for comparison. A bimodal PEC system provides several advantages, such as the long-chain polyelectrolyte is expected to provide a permeable network, while the smaller, multisite, negatively charged polyanion chains are hypothesized to act as molecular connectors, penetrating the inter- and intrachain voids of PDDA and forming multipoint ionic bridges between adjacent chain segments,^25,26^ thereby potentially aiding the construction of an interconnected polymeric network. In addition, it should also be noted that the short-chain polyanions may form multiple ionic connections between different segments of the long polycation chains, thereby distributing these connections.^27,28^ Such an arrangement is expected to improve the mechanical strength per unit mass of the PECs.

Mechanistically, the formation of a PDDA/PPA network, composed of both cationic and anionic polyelectrolytes, involves electrostatic attraction and charge compensation between the two components (see Figure S4 in the Supporting Information for FTIR measurements of the functional group involved). Therefore, the electrophoretic mobility (EPM) of PECs prepared at varying PPA fractions was measured to monitor the evolution of surface charge with composition. If effective electrostatic complexation occurs, the EPM should decrease progressively from positive to near-zero values as the PPA content increases and may even undergo charge reversal, indicating overcompensation of charges.^29–31^ In contrast, weak adsorption would not produce such behavior, suggesting that EPM measurements serve as a critical indicator of effective PDDA–PPA electrostatic interactions.^32,33^

In addition, EPM responses of PECs formed from PDDA with different degrees of polymerization (PDDA_H and PDDA_M) were compared to evaluate the influence of the PDDA scaffold chain length, as characterized by the degree of polymerization, on surface charge evolution and potential network formation.

As shown in Figure 3a, the EPM of PECs decreases overall with an increasing PPA fraction. Although the two systems exhibit distinct declining profiles, both demonstrate progressive compensation of surface positive charges, confirming the effective electrostatic attraction between PDDA and PPA that drives complex formation.

**Figure 3:**
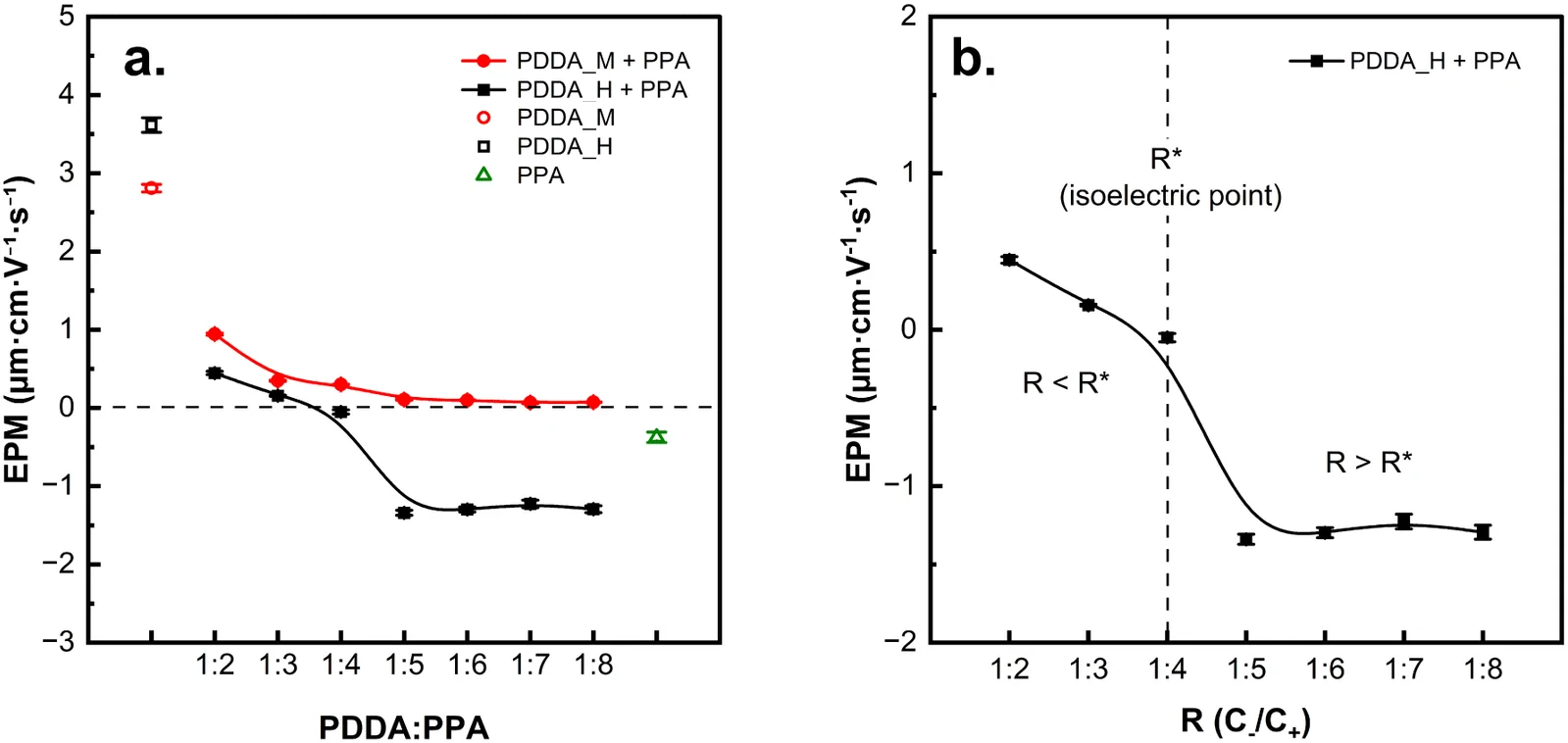
Electrophoretic mobility (EPM) of PECs formed from PDDA and PPA. (a) EPM of PECs prepared from medium- and high-molecular-weight PDDA (PDDA_M and PDDA_H) at different PDDA:PPA monomer ratios; the EPM of the individual components (PDDA_M, PDDA_H, and PPA) is also shown. (b) EPM of PDDA_H/PPA plotted as a function of the monomer ratio *R* = *C*_–_/*C*_+_; the dashed line marks the isoelectric composition R*. The average values and associated standard deviations are based on three data sets.

In the PDDA_M system, as the PPA fraction in the feed increased from 1:2 to 1:8, the EPM continuously decreased from approximately +0.938 μm·cm·V^–1^·s^–1^ at PDDA_M:PPA = 1:2 to about +0.106 μm·cm·V^–1^·s^–1^ at PDDA_M:PPA = 1:5, after which it gradually reached a plateau (Figure 3a, red curve). This trend indicates that the positive charges on PDDA chains are largely neutralized by PPA, thereby limiting further association of additional negatively charged PPA. Notably, charge neutralization occurs at a PDDA_M:PPA monomer molar ratio of 1:5, which is likely because during complexation, the small-sized short-chain PPA molecules are extensively wrapped by, or dispersed within, the much longer PDDA chains, leaving the PECs’ surface predominantly positively charged.

In the PDDA_H system, as the PPA fraction in the feed increased from 1:2 to 1:8, the EPM continuously decreased from approximately +0.520 μm·cm·V^–1^·s^–1^ at PDDA_H:PPA = 1:2 to about −1.193 μm·cm·V^–1^·s^–1^ at PDDA_H:PPA = 1:5, after which it gradually reached a plateau (Figure 3a, black curve). In contrast, the EPM of the PDDA_M system remained positive across the entire composition range, the PDDA_H system not only exhibited EPM values that crossed into the negative regime but also achieved charge neutralization at a lower PPA content, indicating a stronger binding affinity toward PPA.

To better interpret these EPM trends, particularly the more pronounced charge-reversal effect observed for the PDDA_H system, the data for the PDDA_H–PPA complexes were replotted as a function of the monomer molar concentration ratio, defined as *R* = *C*_–_/*C*_+_ (Figure 3b), where *C*_+_ and *C*_–_ denote the monomer-equivalent concentrations of PDDA_H and PPA, respectively. By using a linear stoichiometric charge-neutrality model, assuming that the net charge density is proportional to the difference in charge concentrations:^34^

7 $$Q_{eff}=\alpha C_{+}-\beta C_{-}$$

where α and β denote the effective charge contributions per PDDA and PPA monomer, respectively. Under a linear electrokinetic approximation,^35^ the electrophoretic mobility is proportional to the effective net charge:

8 $$\mu =kQ_{eff}$$

where *k* is a proportionality constant related to hydrodynamic/frictional factors under fixed measurement conditions. Defining the composition variable as

9 $$R=\frac{C_{-}}{C_{+}}$$

the mobility can be expressed as a function of composition:

10 $$\mu \left(R\right)=k\left(\alpha C_{+}-\beta C_{-}\right)=kC_{+}\left(\alpha -\beta R\right)$$

Accordingly, μ­(*R*) decreases overall with increasing *R* and crosses zero at the isoelectric composition R*. From μ­(R*) = 0 (equivalently *Q*_eff_ = 0), we obtain

11 $$R^{*}=\frac{\alpha }{\beta }$$

Thus, the complexes are positively charged for *R* < R* and negatively charged for *R* > R*. Performing this simple analysis on our results in Figure 3b, we found that the observed R* ≈ 4 > 1 implies α > β, indicating that, on a monomer-equivalent basis, each PPA unit is relatively less efficient at neutralizing PDDA positive charges. This explains why a relatively high PPA fraction is required to induce charge reversal.

In addition, the distinct EPM decay profiles of PDDA_M- and PDDA_H- based PECs (Figure 3a) may also arise from the different intrinsic EPM values of PDDA_M and PDDA_H, which are approximately +2.809 and +3.614 μm·cm·V^–1^·s^–1^, thereby reflecting differences in their initial surface charge densities. Furthermore, longer PDDA_H chains tend to facilitate the formation of a more interconnected network upon electrostatic complexation with PPA, which may correspond to a reduced characteristic mesh size.^25,28,36^ When excess, small-sized PPA molecules further penetrate these voids,^37–39^ they may become entangled within the network, enabling the incorporation of more PPA at the same PDDA:PPA ratio and resulting in more negative EPM values. As the PPA content continues to increase, intermolecular electrostatic repulsion begins to dominate, and the “accommodation capacity” of the network approaches saturation. A dynamic balance between adsorption and repulsion is thereby established, leading to the stabilization of EPM values once the PDDA_H:PPA ratio reaches approximately 1:5.

To further probe how PDDA_H/PPA PECs respond to variations in ionic strength, NaCl was introduced to fine-tune the ionic strength to allow systematic adjustment of the electrokinetic properties and dispersed structure of PECs. Here, EPM and hydrodynamic size of the PECs were recorded as a function of salt concentration (Figure 4a,b), together with time-evolution measurements of dispersion stability under different medium conditions (Figure 4c–e). Three representative compositions, PDDA_H:PPA = 1:2, 1:4, and 1:6, were selected because their EPM values in deionized water are positive (EPM > 0), near-neutral (EPM ≈ 0), and negative (EPM < 0), respectively, enabling a clear comparison of salt effects across distinct initial charge states.

**Figure 4:**
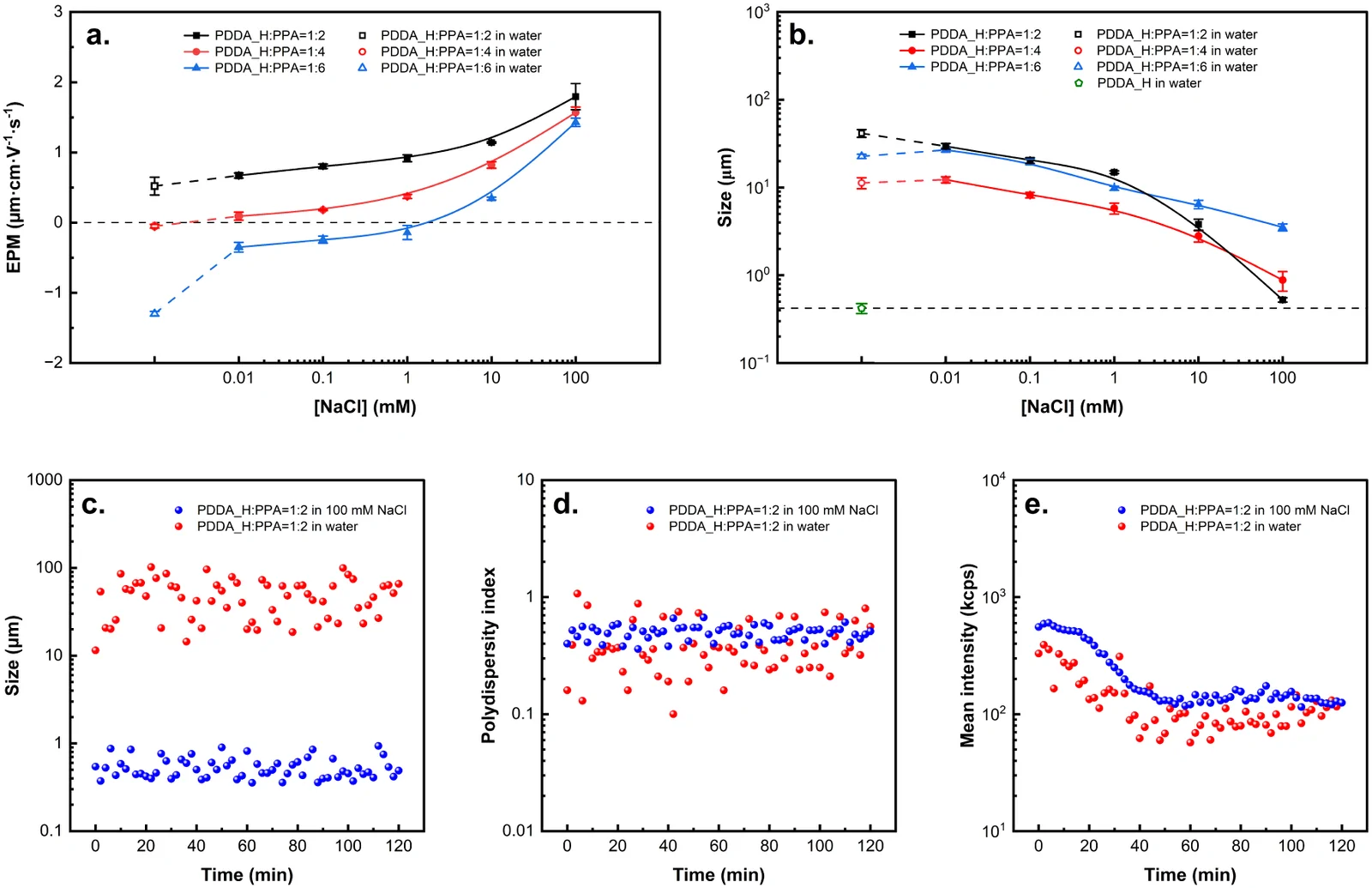
Effect of NaCl ionic strength on the EPM and hydrodynamic size of PDDA_H/PPA PECs. (a) EPM of PECs prepared at PDDA_H:PPA monomer ratios of 1:2, 1:4, and 1:6 as a function of NaCl concentration. (b) Hydrodynamic size (DLS) of the same samples as a function of NaCl concentration. Open symbols denote measurements performed in deionized water (0 mM NaCl), plotted at 0.001 mM NaCl solely for visualization purposes on the logarithmic concentration axis, whereas filled symbols denote measurements in NaCl solutions. The average values and associated standard deviations are based on three data sets. (c–e) Time evolution of the hydrodynamic size (c), polydispersity index (d), and mean scattering intensity (e) of PDDA_H:PPA = 1:2 PECs measured in 100 mM NaCl and in deionized water.

In a conventional electrokinetic framework, if the complex composition and the surface-charge distribution remain unchanged upon salt addition, increasing ionic strength screens electrostatic interactions and the magnitude of EPM is expected to decrease progressively toward zero.^35,40^ But such a framework generally cannot account for mobility sign reversal. As shown in Figure 4a, the EPM values of all three compositions shift systematically in the positive direction with increasing NaCl concentration. This behavior suggests that salt addition in the present system does more than simply compress the electrical double layer; rather, it likely alters the effective charge state of the PECs. A plausible explanation is that elevated ionic strength weakens the ionic associations of PECs formed,^41^ causing partial dissociation/desorption of PPA from PDDA. This would reduce the anionic contribution of PPA at the complex surface and expose more cationic PDDA sites, thereby shifting EPM toward positive values and leading to a change in sign. Collectively, the trends in Figure 4a indicate that these PECs do not behave as electrokinetically “fixed” complexes in saline media but instead exhibit dynamic charge behavior.

Complementing the salt-induced electrokinetic reorganization reflected in Figure 4a, Figure 4b shows a pronounced decrease in hydrodynamic size with increasing NaCl concentration. We consider this size reduction to be most likely associated with a salt-induced weakening of PPA-mediated bridging, because reduced bridging may destabilize larger PEC aggregates and promote their disassembly or fragmentation into smaller dispersed entities, thereby yielding smaller characteristic sizes in DLS.^42,43^ At the same time, an alternative explanation must also be considered, namely that at high ionic strength, larger aggregates sediment preferentially, so that DLS mainly probes the smaller and more stably suspended species in the supernatant, leading to an apparent size decrease.^44–46^ To distinguish between these possibilities, we further examined the temporal stability of PECs in deionized water and in salt solution (Figure 4c–e). As shown in Figure 4c, the PECs in water exhibit a broader size distribution, whereas those in salt solution are more narrowly distributed, consistent with the PDI results in Figure 4d. More importantly, under both medium conditions, neither the characteristic size nor the PDI shows a systematic temporal change over the measurement period, indicating that once formed in each medium, the dispersed structures maintain a relatively stable characteristic size distribution. Meanwhile, Figure 4e shows that the mean scattering intensity in both systems decreases initially and then approaches a plateau. Because the mean scattering intensity is mainly affected by the number and size of particles passing through the optical path,^44,45^ and because Figure 4c indicates no obvious time-dependent change in particle size, the decrease in intensity is more reasonably attributed to a reduction in particle number, i.e., partial sedimentation occurring in both media. Therefore, although sedimentation does occur, it does not lead to a continuous decrease in the measured particle size over time. Accordingly, the size decrease observed in Figure 4b should be attributed primarily to intrinsic restructuring of the PEC dispersion under ionic-strength perturbation, rather than to sedimentation-driven selective sampling.

Taken together, the systematic evolution of the EPM of the PECs with increasing PPA fraction (Figure 3a), along with the salt-dependent changes in EPM and hydrodynamic size (Figure 4a,b), demonstrates that effective electrostatic attraction exists between PDDA and PPA and that the resulting PECs can undergo a certain degree of structural reorganization in saline media. Moreover, the charge neutralization observed only at relatively high PPA fractions, together with the distinct EPM evolution profiles of the PDDA_M and PDDA_H systems (Figure 3a), further suggests that PPA does not merely adsorb on the outer surface of PDDA but is more likely to penetrate the inter- and intrachain voids of PDDA, where it can establish multipoint ionic linkages between adjacent polymer chains or between different segments of the same chain as hypothesized.

To elucidate the PPA–PDDA adsorption process on the silica surface as a sand mimic and the resulting structural characteristics of PECs, particularly the potential formation of a networked structure, QCM-D was employed to monitor the real-time adsorption and assembly of PDDA_H and PPA. Specifically, a baseline for Δ*f* and Δ*D* was established by rinsing with deionized water, followed by alternating injections of PDDA_H (5 min) and PPA (5 min) for five cycles to observe the stepwise construction and evolution of the composite layer (s). Finally, deionized water was used to remove loosely bound components. The results are shown in Figure 5a.

**Figure 5:**
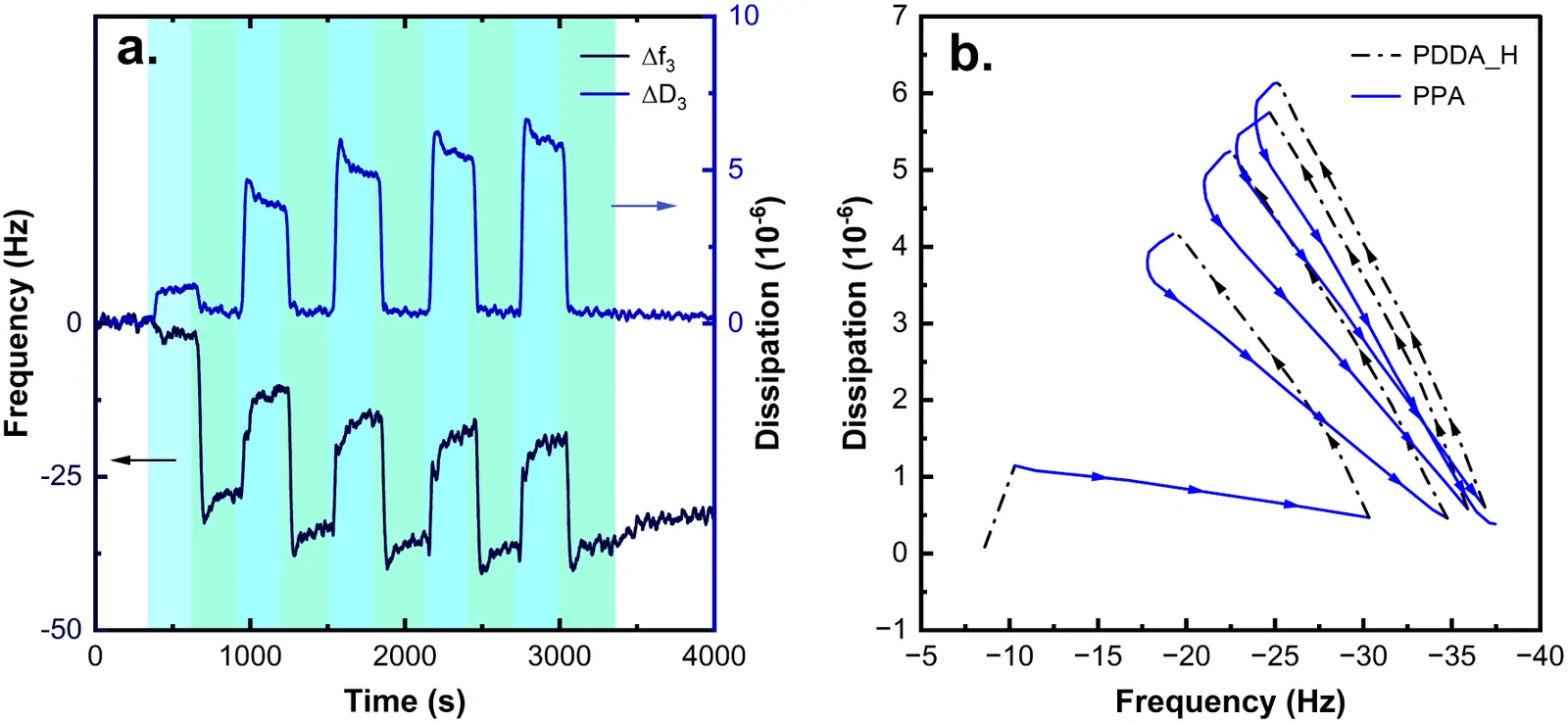
Real-time QCM-D responses for the stepwise assembly of PDDA_H/PPA on SiO_2_ surfaces (third overtone). (a) Time evolution of the frequency shift (Δ*f*_3_, black, left axis) and dissipation change (Δ*D*_3_, blue, right axis). Light blue and light green regions indicate the injection periods of PDDA_H and PPA, respectively, whereas the white regions correspond to rinsing with deionized water (see Figure S6 in the Supporting Information for multiple-overtone data). (b) Δ*D*_3_ versus Δ*f*_3_ plot (dissipation versus frequency shift) for the PDDA_H/PPA layers on the SiO_2_ surface at the third overtone. Arrows indicate the evolution direction of each adsorption step during the sequential injection of PDDA_H and PPA.

During the first PDDA–PPA adsorption cycle, injection of PDDA resulted in a decrease in frequency (Δ*f*_3_) and an increase in dissipation (Δ*D*_3_) (see Figure 5a), indicating the formation of a relatively soft cationic polyelectrolyte layer on the SiO_2_ surface. The amount adsorbed corresponds to 5.12 × 10^–11^ mol of PDDA monomer-equivalent units. Upon subsequent injection of PPA, Δ*f*_3_ decreased further which indicates the subsequent adsorption of PPA onto the preformed PDDA layer. This process is likely driven by electrostatic interaction,^47^ consistent with the significant contrast in electrophoretic mobility (EPM) observed between the two species (a difference of ≈ 4 μm·cm·V^–1^·s^–1^). The adsorbed amount at this stage corresponds to 1.48 × 10^–9^ mol of PPA monomer equivalents, approximately 28.9 times that of PDDA. Concurrently, Δ*D*_3_ decreased, indicating that the initially soft PDDA layer became more rigid after PPA adsorption. This behavior is consistent with the mechanism in which short-chain PPA acts as a “molecular connector”: PPA penetrates the inter- and intrachain voids of PDDA and restricts chain mobility through multipoint electrostatic bridging, thereby creating a compact layered structure.

After the silica-PDDA-PPA-layered structure formed during the first adsorption cycle, a substantial amount of PPA had already accumulated at the interface, forming electrostatic attraction from the inner PDDA layer toward the outer PPA layer that was markedly weakened due to the interscreening effect. Upon the second PDDA adsorption cycle, the incoming PDDA did not immediately overcoat the existing PPA layer; instead, it displaced part of the previously adsorbed PPA through electrostatic interactions before recombining with the remaining PPA. It is possible that the amount of PPA removed exceeded the amount of newly adsorbed PDDA, consequently causing an overall mass reduction, leading to an increase in the QCM-D frequency shift (Δ*f*_3_). Moreover, it should also be noted that a “mass paradox” could occur in the QCM-D measurement due to viscoelastic/hydration changes; specifically, the water trapped in the sandwiched PPA layer was expelled, thereby causing a hydrodynamically coupled mass decrease.^48^ PPA is significantly more hydrophilic than PDDA, because the phosphate groups possess high hydrogen bonding capability and strong hydration strength.^49^ Hence, any structural change such as compaction will remove or decouple a large amount of hydrodynamically coupled water, leading to an apparent mass loss (or frequency increment). At this stage, the film structure can be approximated as a “PDDA–PPA-PDDA” configuration, in which the outermost layer is dominated by relatively soft PDDA, resulting in an increase in dissipation (Δ*D*). On a monomer-equivalent basis, the amount of PPA on the crystal at this point was approximately 4.8 times that of PDDA, consistent with the EPM trend, which plateaued once the PPA content reached roughly five times that of PDDA. This agreement suggests that when the PDDA_H:PPA monomer molar ratio approaches the range of 1:4 to 1:5, the system may reach a balance between charge compensation and spatial filling, forming a relatively stable PEC network structure at the interface. Upon the subsequent injection of PPA, both Δ*f*_3_ and Δ*D*_3_ decreased again, indicating successful adsorption of the new PPA layer onto the outer PDDA and its possible further penetration into the PDDA matrix, thereby densifying the film and enhancing its rigidity.

As shown in Figure 5b, the Δ*D*_3_–Δ*f*_3_ plot at the third overtone also captures several key stages of the PDDA/PPA adsorption process. In every cycle, the Δ*D*_3_/Δ*f*_3_ slope for the PDDA adsorption process is always steeper than its corresponding PPA adsorption process. Physically, this means that for each unit mass adsorbed leading to a frequency shift, the PDDA layer gains more dissipation (more “softness”) compared to PPA. Such a trend can be explained by (1) PDDA forms a more extended soft brush-like layer whereas PPA serves as a cross-linker to connect the PDDA segments leading to interlayer compaction (as discussed in Figure 3), (2) the sandwiching effect that releases coupled water molecules, and (3) PPA is more likely to collapse onto the PDDA layer due to charge compensation leading to densification. Moreover, with an increasing number of PDDA/PPA adsorption cycles, the slope of the Δ*D*_3_/Δ*f*_3_ trajectory during each PPA injection gradually increases, indicating a progressively stronger modulation of film softness (or rigidity) by PPA and a continuous densification of the layer(s). Based on this observation, we infer that in practical sand-stabilization applications, applying PPA in multiple small doses rather than in a single large addition may be more effective in promoting the formation of a tighter, more shear-resistant PECs network, thereby enhancing the sand-stabilization performance.

Finally, the decrease in EPM (and the charge reversal observed in the PDDA_H/PPA system; Figure 3a) with increasing PPA content confirms the presence of effective electrostatic attraction between PPA and PDDA. Moreover, the isoelectric point occurs at an unusually high PPA fraction (R* ≈ 4, corresponding to PDDA_H:PPA ≈ 1:4; Figure 3b), and the NaCl perturbation experiments show that with increasing NaCl concentration, the EPM shifts overall in the positive direction while the hydrodynamic size decreases markedly (Figure 4). Together with the simultaneous decreases in both Δ*f*_3_ and Δ*D*_3_ observed in the QCM-D measurements (Figure 5), these results collectively support our molecular connectors hypothesis.

### 3.2 Network Strength and Rigidity of PDDA/PPA PECs Probed by Rheology

To further evaluate how flow shearing influences the strength and stability of the PEC network, a series of PDDA–PPA complexes were prepared by systematically varying the PPA fraction and the PDDA molecular weight (PDDA_M vs PDDA_H), while maintaining a constant PDDA molar composition. The resulting assembled layers were then examined by using oscillatory rheology.

In this work, frequency sweep measurements were conducted at a fixed strain amplitude of γ_0_ = 50%, and the angular frequency *ω* was converted into an effective shear rate γ̇ = γ_0_ω. Because of our goal is to relate the imposed deformation, which is kinematics in nature, rather than the material response, this expression is used to reflect both the “rate” and the “intensity” of external shear perturbation. At a constant strain amplitude, a larger γ̇ corresponds to more loading–unloading cycles imposed on the PEC network per unit time, faster completion of deformation, and consequently less time available for ionic associations and chain rearrangements to “self-repair”.^50,51^ Thus, γ̇ can be regarded as an external variable that controls the extent of shear-induced damage, in which a higher γ̇ signifies more severe shear disturbance and stronger network disruption. Meanwhile, the storage modulus *G*′, multiplied by the strain amplitude (τ ≈ *G*′γ_0_), represents the characteristic elastic shear stress that the network can sustain at a given shear rate. If the PECs network remains largely intact at a given γ̇, the system can support a relatively high elastic stress; once parts of the interconnected structure are broken by shear, the load-bearing capacity decreases, resulting in reductions in both *G*′ and τ. Accordingly, in the subsequent data analysis, γ̇ is used to quantify the “damage intensity” imposed by external shear on the PEC network, whereas the stress scale derived from *G*′(τ) is used to characterize the elastic load-bearing capacity that the network can still sustain under that imposed damage intensity. Together, these two metrics provide a quantitative framework for comparing the strength and shear stability of the polymeric network structures formed in PDDA/PPA PECs with different compositions.

As shown in Figure 6a, in the PDDA_M system, the *G*′ of the blank PDDA_M sample (without PPA) drops drastically even with a slight increase in frequency, retaining elasticity only in the low-frequency region. This behavior is characteristic of weakly cross-linked polyelectrolyte, hinting a dynamically bonded polymer networks, especially those held together by electrostatic pairings.^52^ This result indicates that PDDA_M alone is readily “disrupted” under shear and is unable to form a stable supporting network. As the composition of PPA increases, the resulting PECs exhibit a pronounced increase in *G*′ (corresponding to enhanced network strength), and the elevated *G*′ values are maintained over a broader frequency range. Here, increasing PPA strengthens the polyelectrolyte networks formed by increasing the density of ionic bridging. When the PDDA_M:PPA ratio exceeds 1:6, the increase in *G*′ becomes nearly saturated, indicating that the available PDDA sites capable of being effectively bridged by PPA are approaching their limit, and further addition of PPA no longer leads to substantial enhancement of the PECs network strength.

**Figure 6:**
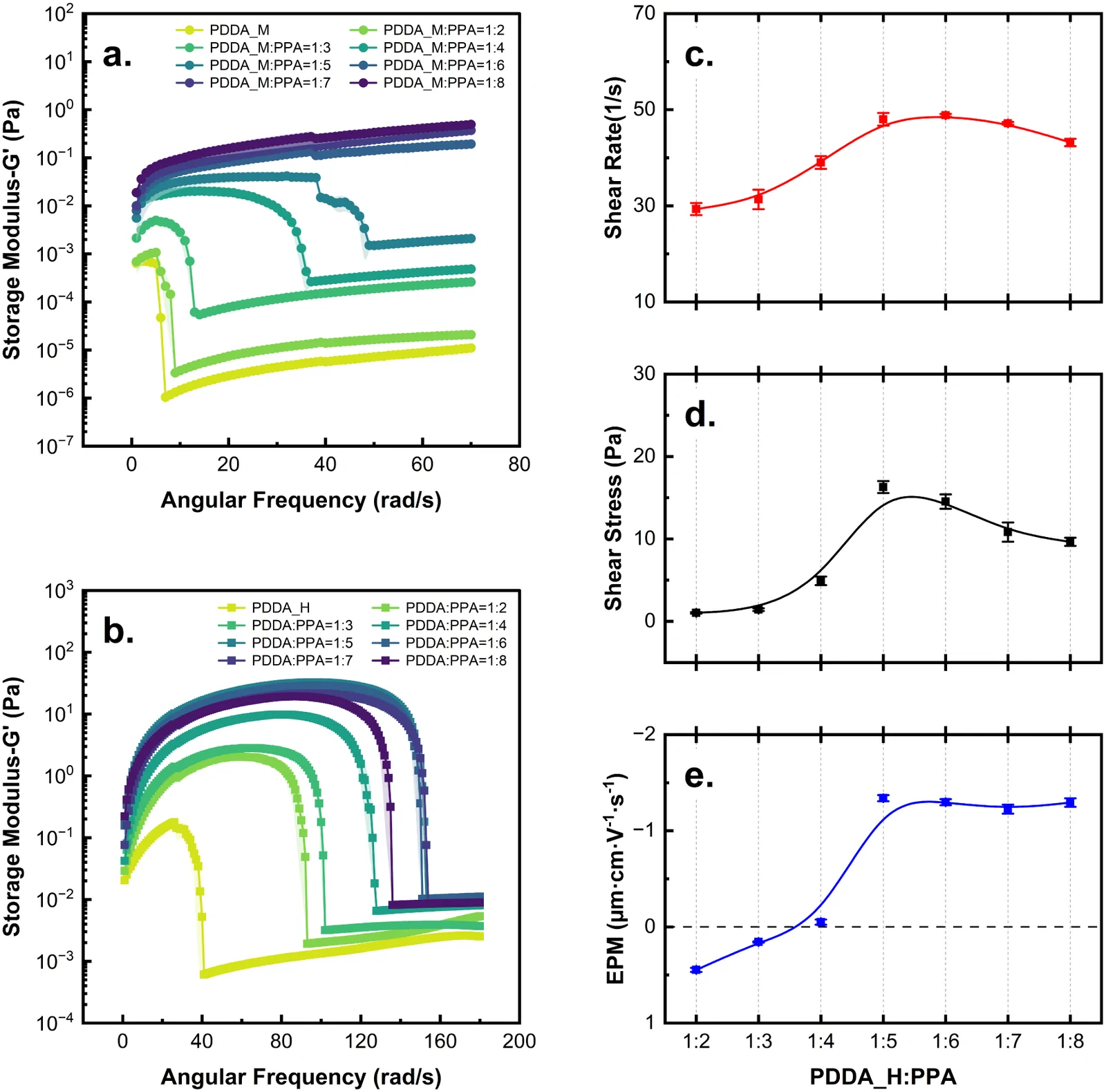
Rheological responses of PECs with different PDDA/PPA compositions. (a) Frequency-dependent storage modulus *G*′ of PECs formed from PDDA_M with varying PPA fractions. (b) Frequency-dependent storage modulus *G*′ of PECs formed from PDDA_H with varying PPA fractions. Shaded bands represent the standard deviation. (c) For the PDDA_H system, effective shear rate γ̇ obtained by converting the angular frequency at which *G*′ reaches its maximum for each composition. (d) For the PDDA_H system, characteristic shear stress τ calculated from the maximum *G*′ at each composition. (e) Electrophoretic mobility (EPM) of PDDA_H:PPA PECs at different molar ratios. The average values and associated standard deviations are based on three data sets.

As shown in Figure 6b, in the PDDA_H system, the *G*′ values of the PDDA_H-PPA PECs are consistently higher than those of the PDDA_M-PPA system across all PPA fractions, indicating that longer PDDA_H chains are more effective in forming stiffer PECs networks because they provide more segments that can be bridged by PPA to form multivalent cross-links. For each PDDA_H:PPA ratio, the maximum storage modulus $G_{max}^{’}$ and its corresponding angular frequency ω_max_ were extracted and converted to the effective shear rate γ̇ = γ_0_ω_max_ and the characteristic shear stress $\tau \approx G_{max}^{’}\gamma_{0}$ , which are plotted in Figure 6c and d, respectively. Both quantities increase with increasing PPA content, reach their highest values at PDDA_H:PPA = 1:5, and then slightly decrease thereafter. Notably, the rheological optimum is higher than the isoelectric composition obtained from EPM analysis (R* ≈ 4 corresponding to PDDA_H:PPA ≈ 1:4), placing it on the mildly PPA-rich side. While R* marks the composition at which the complexes are electrically neutral, PDDA_H:PPA = 1:5 corresponds to an overcompensated state (*R* > R*). Under this condition, the density of PDDA_H-PPA bridging points reaches its maximum, and the network stiffness is consequently the strongest. When the PPA content exceeds 1:5, however, the excess anionic PPA introduces substantial electrostatic repulsion, weakening the existing PDDA_H-PPA associations and gradually loosening the network structure.

As shown in Figure 6e, the evolution of EPM in the PDDA_H:PPA system closely mirrors that of the rheological response. At a PDDA_H:PPA ratio of 1:5, the EPM curve approaches a plateau, coinciding with the maxima in rheological parameters that characterize the network stiffness and resistance to shear-induced damage. This observation suggests that 1:5 is not only the mechanically optimal composition for the PEC network formed but also represents a composite equilibrium state governed by multiple competing interactions. At this ratio, a relatively stable equilibrium is established among three factors: (i) the amount of PPA incorporated into the PEC network, (ii) the attractive electrostatic binding between PDDA and PPA, and (iii) the repulsive forces between PPA chains. The QCM-D results (see Figure 5) further support this interpretation in which after one full adsorption cycle on the crystal surface, the monomer molar ratio of PDDA to PPA remaining in the assembled PEC layer is likewise close to 1:5, corroborating the significance of this equilibrium composition.

To evaluate whether short-chain PPA molecules can access the internal voids of the long-chain PDDA network, we estimated the effective elastic mesh size of the PDDA network, ξ_el_, from the plateau storage modulus *G*′ as shown in eq 12,^53^

12 $$\xi_{el}\approx {\left(\frac{k_{B}T}{G^{’}}\right)}^{1/3}$$

where *k*_B_ is the Boltzmann constant (1.38 × 10^–23^ J·K^–1^), *T* is the absolute temperature (298 K), and *G*′ is the maximum storage modulus of the pure PDDA_H sample extracted from the frequency sweep shown in Figure 6b, with *G*′ = 0.18 Pa. The underlying physics implied by this expression is that the penetration of PPA molecules into the PDDA network is mainly driven by Brownian motion, and this thermally driven tendency to diffuse is balanced by the elastic shear stiffness of the PDDA network. The resulting length scale ξ_el_ thus reflects a normalized mean free void size of the PDDA system at room temperature, rather than a fixed length scale of the pore size. Using this approach, ξ_el_ was calculated to be approximately 284 nm. In contrast, the characteristic size of a PPA molecule is much smaller. According to the molecular modeling results in Supporting Information S2.2, an isolated PPA chain with a degree of polymerization of 6 exhibits a maximum interatomic separation of approximately 0.88 nm in its optimized conformation. The large disparity between ξ_el_ and the PPA molecular length indicates that PPA chains are small enough to thermally diffuse through the inter- and intrachain voids of the PDDA network.

Overall, two rheological parameters were used to characterize the strength and stability of the PEC network, namely the shear rate γ̇, which reflects the “damage intensity” imposed on the PECs, and the shear stress τ, which represents the stability of the PEC network under a given shear condition. The results show that an appropriate amount of PPA significantly enhances the stability of the PEC network, with both parameters reaching their maximum values at a PDDA_H:PPA ratio of 1:5. This optimal ratio is also supported by the EPM data (Figure 6e) and is again consistent with the QCM-D measurements, suggesting that the 1:5 composition arises from a balance among multiple competing interactions.

### 3.3 Effect of PECs on Sand Aggregation under Hydrodynamic Shear

We have quantified the structural stability of PECs using oscillatory rheology and established how the PDDA chain length and the PPA fraction influence the strength of the PEC network formed. To further quantify the role of the PDDA-to-PPA ratio in dictating the physical properties of PECs in sand stabilization, PECs with different compositions were mixed with sand particles and subjected to rotational shearing tests using the custom-built apparatus shown in Figure 2. The critical rotational speed at which each sand cluster underwent complete disintegration was recorded and converted into the corresponding effective shear rate and shear stress using eqs 5 and 6, thereby providing a quantitative measure of the mechanical stability of the sand cluster formed under varying degrees of hydrodynamic shear.

Across all compositions, PECs formed from PDDA_M and PPA exhibited weak sand-binding and aggregation capabilities, making it difficult to form sand clusters that were stable enough for mechanical testing; thus, these samples could not be evaluated. In the PDDA_H system, samples with insufficient PPA (e.g., PDDA_H:PPA = 1:1) also failed to produce a consolidated sand-PEC cluster, and therefore, this ratio was likewise excluded from subsequent mechanical tests.

As shown in Figure 7a, the maximum rotational speed that the sand clusters can withstand before disintegration increases with increasing PPA amount, reaches a maximum at a PDDA_H:PPA ratio of 1:5, and then gradually decreases. This trend is also reflected in the visible residual sand-cluster area after shearing, as shown in Figure 7a (see Supporting Information S7 for full time-lapse images at various PDDA/PPA ratios). To allow direct comparison with the rheological analysis, these critical rotational speeds were converted into equivalent shear rates and shear stresses. Their variations with the PDDA_H:PPA ratio are presented in Figure 7b and c. The equivalent shear rate and shear stress obtained from the mechanical shearing tests exhibit trends that closely mirror the rheological results: both parameters increase steadily as the PPA fraction rises from 1:2 to 1:5, peak at 1:5, and subsequently decrease slightly. These results indicate a clear positive correlation between the strength of the PEC network regulated by PPA and the resistance of the sand clusters to hydrodynamic disintegration, supporting our hypothesis that the PEC network provides structural reinforcement governing sand aggregation and cluster stability. Notably, the PDDA_H:PPA = 1:5 ratio corresponds to the maximum shear rate and shear stress observed in both mechanical tests and rheological measurements. Moreover, both EPM and QCM-D results also converge toward this composition. Together, these observations suggest that the PEC network formed at the 1:5 ratio represents a balanced state arising from multiple competing interactions, yielding a denser and more stable structure. The apparent excess of PPA at this optimal ratio may be attributed to the chain-length distribution of PPA. PPA is a mixture of polyelectrolytes with different chain lengths,^15^ and not all chains are sufficiently long to function effectively as “connectors”. Therefore, we hypothesized that only PPA chains above a certain length threshold can span the relatively large voids (see Brownian calculations in the previous section) in the PDDA network and form effective bridges between two PDDA chains.

**Figure 7:**
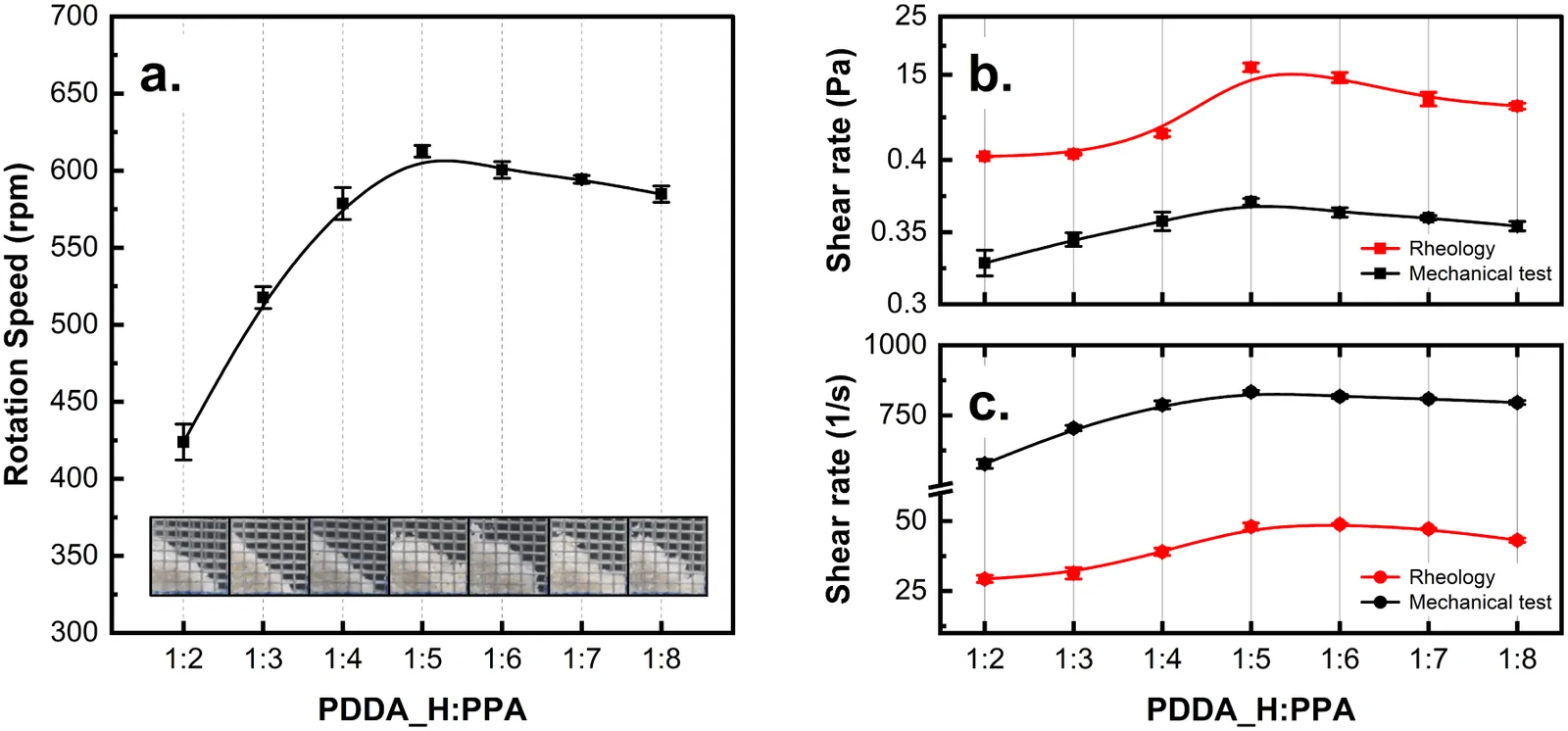
Mechanical stability of sand clusters and its correlation with rheological properties. (a) Critical rotational speed *n*_c_ of sand clusters composed of PECs with different PDDA_H:PPA molar ratios obtained from rotational shearing tests. The attached image sequences show the residual sand remaining in the mesh basket after shearing for each composition. (b) Comparison between the characteristic shear stress converted from the critical rotational speed (black, mechanical testing) and the characteristic shear stress obtained from rheological measurements (red). (c) Comparison between the effective shear rate converted from the critical rotational speed (black, mechanical testing) and the effective shear rate determined from rheological measurements (red). The average values and associated standard deviations are based on three data sets.

### 3.4 Microscopic Evidence of PDDA/PPA-Induced Sand Aggregation Revealed by SEM/EDX

To compare changes in sand aggregation before and after PDDA/PPA treatment, SEM was used to observe the particle morphology, while EDX was employed to analyze the elemental distributions and relative contents of C, N, P, and Si.

The untreated sand particles were randomly dispersed, with no obvious aggregation observed, and their surfaces showed distinct layered textures and sharp protrusions. In contrast, the sand particles treated with PDDA/PPA PECs exhibited obvious aggregation and a smoother surface morphology (Figure 8a–d). Quantitative EDX analysis of the region shown in Figure 8b revealed the presence of Si, C, N, and P in the treated sand sample, with atomic percentages of 8.43%, 20.77%, 2.01%, and 7.36%, respectively. The N:P atomic ratio was approximately 1:3.7, reflecting the relative composition of PDDA and PPA in this region, but differing from the initial feed ratio of 1:5. This difference may be related to the local randomness of the EDX analysis region and the heterogeneity of the sample surface composition.

**Figure 8:**
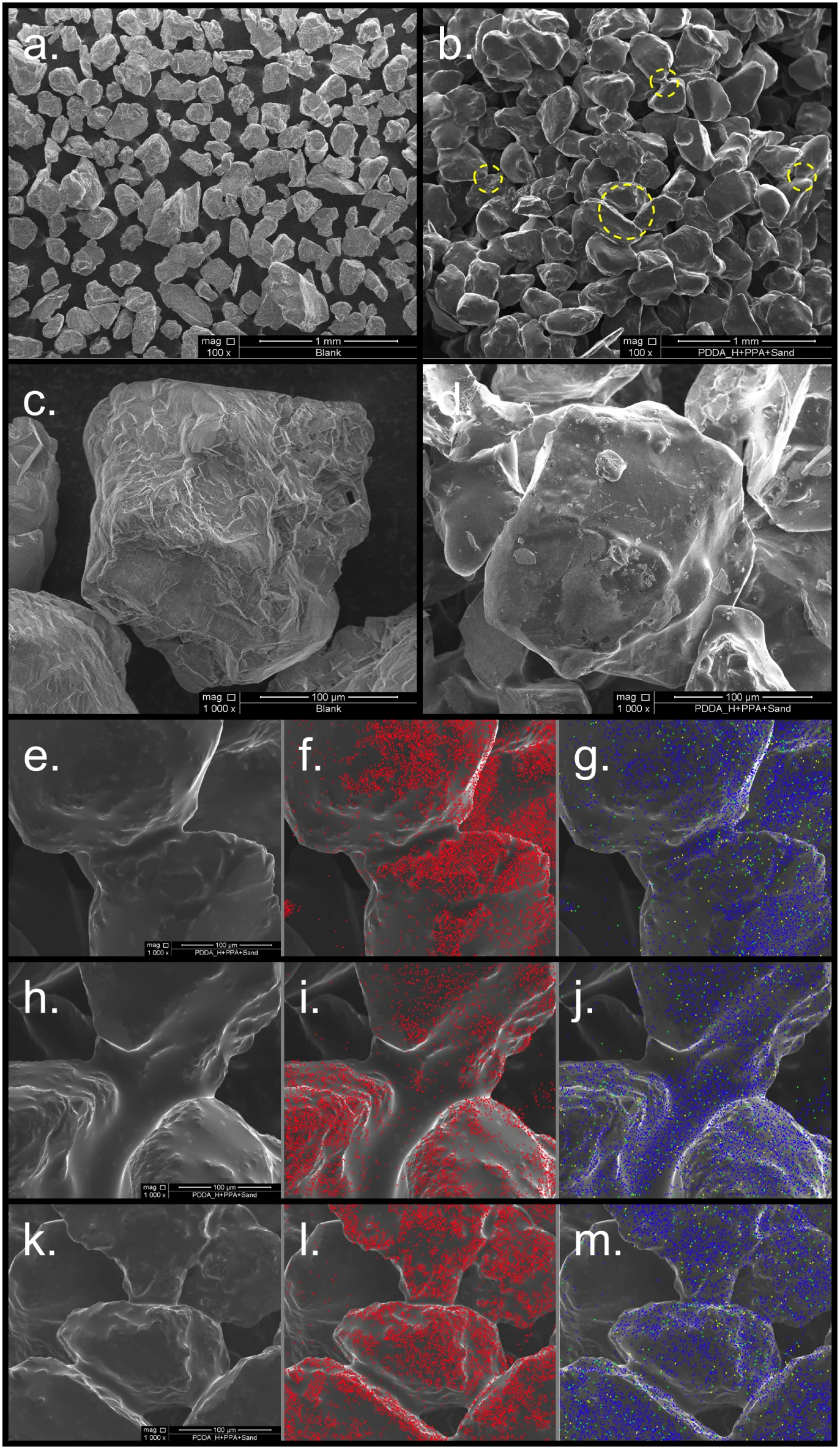
SEM/EDX characterization. (a) Untreated sand, SEM image, 100×. (b) PDDA_H/PPA-treated sand, SEM image, 100×. (c) Untreated sand, SEM image, 1000×. (d) PDDA_H/PPA-treated sand, SEM image, 1000×. (e, h, k) High-magnification SEM images of representative interparticle bridging regions in PDDA_H/PPA-treated sand, 1000×. (f, i, l) Corresponding EDX elemental maps of Si (red). (g, j, m) Corresponding EDX elemental maps showing the distribution of C (green), N (yellow), and P (blue).

In Figure 8b, several distinct bridging materials can be observed between adjacent sand particles and are colored yellow. We further performed EDX elemental mapping of these bridging regions. The results showed that the Si signal was almost absent in the bridging regions (Figure 8f,i,l), thereby excluding the possibility that this material originated from quartz itself. In contrast, N, C, and P signals were observed in these regions (Figure 8g,j,m), corresponding to the characteristic elements of PDDA and PPA. Overall, the SEM/EDX results support the idea that PDDA/PPA PECs promote sand aggregation through both surface coating and interparticle bridging.

## 4 Conclusions

This study investigates the mechanism governing polyelectrolyte complexes (PECs) formed from long- and short-chain polyelectrolytes for sand-stabilization applications and proposes a molecular connector model as the core action pathway.

The findings suggest that the short-chain anionic polymer PPA does not simply adsorb onto the long-chain cationic polymer PDDA; instead, it may penetrate the polymer framework and act as an electrostatic bridge that cross-links PDDA chains into a dense, mechanically robust network. Multiple lines of evidence support this conclusion, including (i) scanning electron microscopy coupled with energy-dispersive X-ray spectroscopy (SEM-EDX) providing direct visualization of the network formed as physical bridges connecting sand grains, (ii) quartz crystal microbalance with dissipation (QCM-D) measurements confirming that the incorporation of PPA renders the polymer layers more compact and rigid, and most importantly, (iii) the strong agreement between macroscopic hydrodynamic erosion tests and microscopic rheological data suggesting that the strength of the ionically bridged polymeric network is an important factor influencing sand-stabilization performance. Collectively, these findings suggest that the ionically bridged polymer network plays an important role in the stabilizing effect of PECs and is closely associated with an improved sand-stabilization performance. This work therefore highlights the importance of considering internal structural engineering of load-bearing networks in addition to surface-level interactions in the design of polymer-based stabilizers. Beyond sand management, the proposed concept may provide a transferable framework for designing robust soft matter systems, including hydrogels and functional coatings, where mechanical integrity can be enhanced through controlled network architecture.

## Supplementary Material



## Notes

Rationale for using a monomer-equivalent basis in PDDA/PPA composition calculations (Section S1); calculation of PECs dosage for sand aggregation (Section S2); determination of the drag coefficient *C*_d_ (Section S3); control experiments of single-polyelectrolyte systems and PDDA-based PEC systems (Section S4); FT-IR characterization of PDDA/PPA polyelectrolyte complexes (Section S5); multiple-overtone QCM-D responses during the alternating adsorption of PDDA_H and PPA on SiO_2_ surfaces (Section S6); time-resolved photographic snapshots of the mechanical stability test (extracted from video recordings) (Section S7) (PDF)
