# Supplementary material for: Network Formation in Poly(diallyldimethylammonium chloride)/Polyphosphoric Acid Polyelectrolyte Complexes and Their Role in Sand Stabilization
Source: Langmuir. 2026 Aug 31;42(36):26275–89. doi: 10.1021/acs.langmuir.6c02727 (PMC13585129; doi:10.1021/acs.langmuir.6c02727)
Supplement: Supplementary file 1 [file la6c02727_si_001.pdf]

Supporting Information for

Network Formation in Poly(diallyldimethylammonium chloride)/Polyphosphoric Acid Polyelectrolyte Complexes and Their Role in Sand Stabilization

Rui Zhi Liu[†], *Kong Teng Ling*[‡], *JitKang Lim*[*,†]

[†]School of Chemical Engineering, Universiti Sains Malaysia, 14300 Nibong Tebal, Penang, Malaysia.

[‡]Schlumberger (M) Sdn Bhd, 50450 Kuala Lumpur, Malaysia.

[*] *Corresponding author. Email: chjitkangl@usm.my*

Overview of the Supporting Information

S1. Rationale for Using a Monomer-Equivalent Basis in PDDA/PPA Composition Calculations
S2. Calculation of PECs Dosage for Sand Aggregation
S3. Determination of the drag coefficient C_d
S4. Control experiments of single-polyelectrolyte systems and PDDA-based PECs systems
S5. FT-IR Characterization of PDDA/PPA Polyelectrolyte Complexes
S6. Multiple-Overtone QCM-D Responses during the Alternating Adsorption of PDDA_H and PPA on SiO_2 Surfaces
S7. Time-Resolved Photographic Snapshots of the Mechanical Stability Test (Extracted from Video Recordings)

S1. Rationale for Using a Monomer-Equivalent Basis in PDDA/PPA Composition Calculations

All PDDA/PPA compositions and dosage calculations in this work are reported on a monomer-equivalent basis, rather than on a "polymer-chain molar" basis, for the following reasons:

Why not a chain-molar basis: it relies on an average molecular weight and can introduce systematic errors. PDDA_H exhibits a molecular-weight distribution rather than a single, well-defined chain length. When composition is defined in terms of "moles of polymer chains," the weighed mass must be converted to chain moles using an average molecular weight (e.g., the nominal M_w of PDDA_H, ~4.5×10^5 $g{\cdot}mol^{-1}$). This conversion implicitly assumes that all chains possess the same molecular weight equal to the average value. In reality, if the distribution is skewed toward shorter chains (lower molecular weight), a given mass contains substantially more polymer molecules than estimated from the average molecular weight (and vice versa), reducing quantitative accuracy and comparability across samples or batches.

Why a monomer-equivalent basis is appropriate: PECs stoichiometry is governed by the number of charged monomer units. Each PDDA monomer unit carries one cationic site, whereas PPA provides the corresponding anionic sites. Therefore, charge compensation, component excess, and charge inversion in PECs are fundamentally determined by the population of charged monomer units, rather than by the number of polymer molecules (chains). A monomer-equivalent basis thus more directly reflects the number of electrostatic pairing and bridging sites available in the mixture, enabling a clearer correlation between composition and PECs structure/performance.

How the monomer-equivalent ratio is defined and calculated: converting weighed masses to monomer-equivalent moles. Throughout this work, the “molar ratio” PDDA:PPA refers to the monomer-equivalent molar ratio, i.e., the ratio between the moles of PDDA monomer units and the moles of PPA monomer units. The monomer-equivalent moles are calculated as:

$$n_{\mathrm{PDDA}} = \frac{m_{\mathrm{PDDA}}}{M_{\mathrm{monomer,PDDA}}} \tag{1}$$

where $M_{\mathrm{monomer,PDDA}}$ is the molar mass of the PDDA monomer unit, taken as 161.1 g·mol^{-1} in this work.

$$n_{\mathrm{PPA}} = \frac{m_{\mathrm{PPA}}}{M_{\mathrm{monomer,PPA}}} \tag{2}$$

where $M_{\mathrm{monomer,PPA}}$ is the molar mass of the PPA monomer unit, taken as 80.0 g·mol^{-1} in this work.

S2. Calculation of PECs Dosage for Sand Aggregation

In this section, the total sand surface area is first estimated (Section S2.1), and then a lower-bound PECs dosage required for geometric surface coverage is calculated and compared with the experimental dosage (Section S2.2) to verify that the applied PECs amount is sufficient to cover the sand surface.

S2.1 Calculation of the Total Surface Area of Sand

Sand grains were approximated as spherical particles with an average radius r_{s}. The surface area and volume of a single sand grain are given by:

$$A_{\mathrm{s,single}} = 4\,\pi\,r_{\mathrm{s}}^2 \tag{3}$$

$$V_{\mathrm{s,single}} = \frac{4}{3}\,\pi\,r_{\mathrm{s}}^3 \tag{4}$$

where $A_{\mathrm{s,single}}$ is the surface area of a single sand grain and $V_{\mathrm{s,single}}$ is the volume of a single sand grain. The mass of a single grain is:

$$m_{\mathrm{s,single}} = \rho_{\mathrm{s}}\,V_{\mathrm{s,single}} \tag{5}$$

where $m_{\mathrm{s,single}}$ is the mass of a single sand grain and ρ_{s} is the sand density. For a total sand mass of $M_{\mathrm{s,total}}$, the total number of sand grains is:

$$N_{\mathrm{s,total}} = \frac{M_{\mathrm{s,total}}}{m_{\mathrm{s,single}}} \tag{6}$$

and the total surface area of sand is:

$$A_{\mathrm{s,total}} = N_{\mathrm{s,total}}\,A_{\mathrm{s,single}} \tag{7}$$

In this work, $M_{\mathrm{s,total}}$ = 1.00 g (i.e., 1.00 × 10^{-3} kg), r_{s} = 1.27 × 10^{-4} m, and ρ_{s} = 2.65 × 10^{3} Kg m^{-3}.[1] Substitution gives $A_{\mathrm{s,single}}$ = 2.02 × 10^{-7} m^2, $V_{\mathrm{s,single}}$ = 8.53 × 10^{-12} m^3, $m_{\mathrm{s,single}}$ = 2.26 × 10^{-8} kg, $N_{\mathrm{s,total}}$ = 4.43 × 10^{4}, and thus $A_{\mathrm{s,total}}$ = 8.93 × 10^{-3} m^2.

S2.2 Calculation of the Minimum PECs Dosage for Surface Coverage

Based on the total sand surface area $A_{\mathrm{s,total}}$ obtained in Section S2.1, a molecular-scale surface-coverage estimate was performed to calculate the minimum amount of PECs required for full surface coverage.

A representative PECs structural unit was defined as a combination of one PDDA monomer and one PPA unit (Figure S1a). This structural unit was constructed in Chem3D and geometry-optimized using the MM2 force field, with the Minimum RMS Gradient set to 0.0100. After optimization, the maximum interatomic separation in the optimized conformation, namely the farthest-atom distance, was measured and defined as the effective diameter, d_p, which was determined to be 9.7 Å. In addition, an isolated PPA chain with a degree of polymerization of 6 was also modeled (Figure S1b), and the farthest-atom distance in its optimized conformation was similarly measured to represent the characteristic size of the PPA chain, giving a value of 8.8 Å.

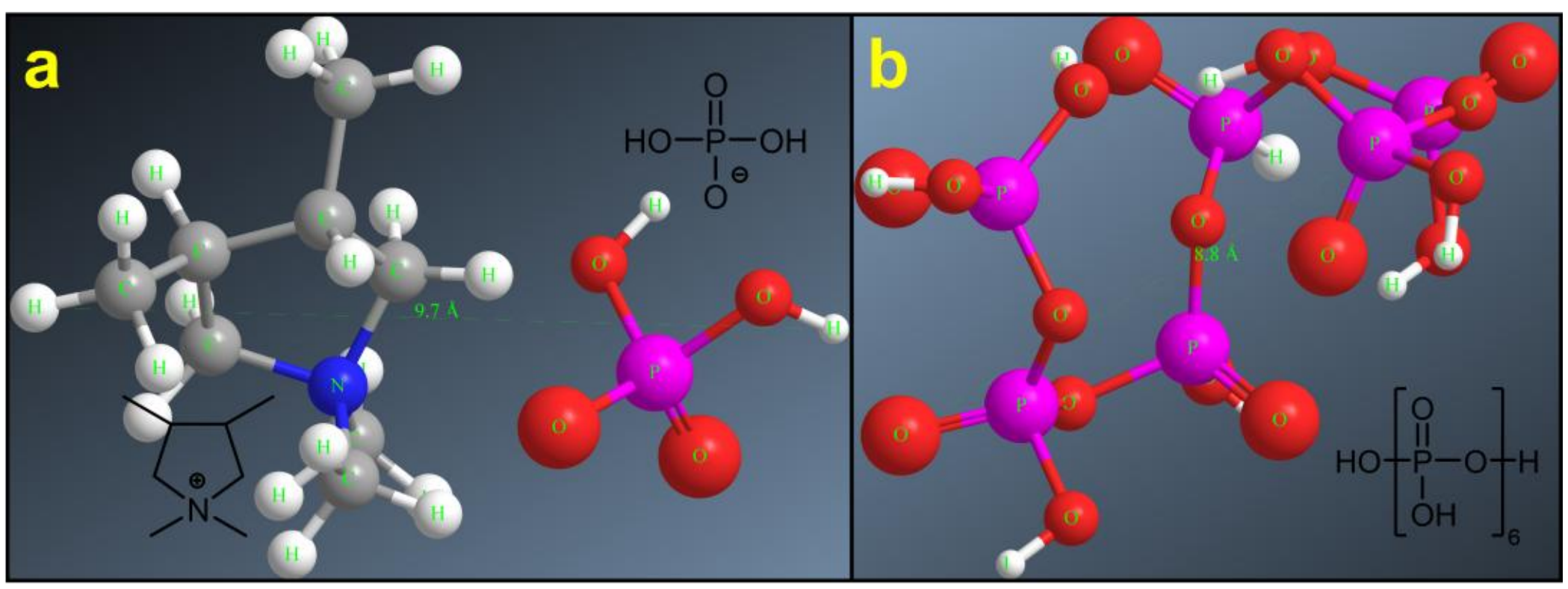

Figure S1. Chem3D models of (a) a representative PECs unit, defined as one PDDA monomer plus one PPA unit, and (b) an isolated PPA chain with a degree of polymerization of 6. The dashed line indicates the maximum interatomic separation (farthest-atom distance), giving a value of 9.7 Å for the PECs unit and 8.8 Å for the PPA chain.

The projected coverage area of one PECs unit is approximated as a circle equivalent (spherical approximation in 3D) with diameter d_p:

$$A_{\mathrm{p,proj}} = \pi \left(\frac{d_\mathrm{p}}{2}\right)^2 \tag{8}$$

The number of PECs units required to cover the sand surface area is:

$$N_{\mathrm{p}} = \frac{A_{\mathrm{s,total}}}{\eta A_{\mathrm{p,proj}}} \tag{9}$$

where η is an areal packing factor ($0 < \eta \leq 1$) accounting for interstitial voids between adjacent PECs units on the surface. The corresponding amount (in moles) is:

$$n_{\mathrm{p}} = \frac{N_{\mathrm{p}}}{N_{\mathrm{A}}} \tag{10}$$

where N_A is the Avogadro constant (6.022×10^{23} mol^{-1}).[2] Because one PECs unit is defined as one PDDA monomer plus one PPA unit, its molar mass is:

$$M_{\mathrm{p}} = M_{\mathrm{monomer,PDDA}} + M_{\mathrm{monomer,PPA}} \tag{11}$$

Thus, the minimum PECs mass required for full surface coverage is:

$$m_{\mathrm{p,min}} = n_{\mathrm{p}}\, M_{\mathrm{p}} \tag{12}$$

For comparison with the experimental dosage, an excess factor is defined as:

$$\text{Excess factor} = \frac{m_{\mathrm{active}}}{m_{\mathrm{p,min}}} \tag{13}$$

In this work, $A_{\mathrm{s,total}}$ = 8.93×10^{-3} m^2 (section S2.1). d_p = 9.7 Å, η = 0.74,[3] $M_{\mathrm{monomer,PDDA}}$ = 161.1 g mol^{-1}, and $M_{\mathrm{monomer,PPA}}$ = 80.0 g mol^{-1} (thus M_{p} = 241.1 g mol^{-1}). Substitution gives $A_{\mathrm{p,proj}}$ = 7.39×10^{-19} m^2, N_{p} = 1.63×10^{16}, n_{p} = 2.71×10^{-8} mol, and $m_{\mathrm{p,min}}$ = 6.53×10^{-6} g. For PECs (PDDA:PPA = 1:5), when mixed with 1 g of sand, 0.2410 g of PECs aqueous solution was added. The solution contained 0.0322 g PDDA and 0.0800 g PPA, corresponding to a total active polymer mass of 0.1122 g (m_{active}

= 0.1122 g). corresponding to an excess factor of 1.72×10^4 relative to the minimum required mass.

Therefore, the applied PECs dosage is more than sufficient to cover the sand surface area in a geometric sense.

S3. Determination of the drag coefficient C_d

In the mechanical stability tests, the critical rotational speed n_c(rpm) corresponding to complete disintegration of the sand cluster was first recorded. The critical Reynolds number Re was then calculated from n_c, and the associated drag coefficient C_d was determined using the standard C_d-Re correlation. The Reynolds number is defined as:

$$Re = \frac{\rho\,(\omega_c r_{cl})\,(2R_s)}{\mu} \tag{14}$$

where ρ is the fluid density, $\mu$is the dynamic viscosity, ω_c is the critical angular velocity, r_{cl} is the distance from the impeller rotation axis to the center of the sand cluster, and R_s is the cluster radius (thus $2R_s$ is the characteristic length scale).

The critical angular velocity ω_c was converted from the critical rotational speed n_c (rpm) as:

$$\omega_c = \frac{2\pi n_c}{60} \tag{15}$$

Substituting Eq. 15 into Eq. 14 yields:

$$Re = \frac{\rho\left(\frac{2\pi n_c}{60} r_{cl}\right)(2R_s)}{\mu} = \left(\frac{\rho\, 2\pi\, r_{cl}(2R_s)}{60\mu}\right) n_c \tag{16}$$

In this work, r_{cl} = 6.5 cm (i.e., 6.5 × 10^{-2} m) and R_s = 0.5 cm (i.e., 5.0 × 10^{-3} m). Water properties at 25 °C were used: ρ = 997 kg m^{-3}, μ = 0.89 mPa•s (i.e., 8.9 × 10^{-4} Pa·s). Substitution gives:

$$Re = 76.25\, n_c \tag{17}$$

After obtaining Re, the drag coefficient C_d was determined from the standard C_d–Re correlation for a rough sphere (Figure S2).[4] The critical rotational speed n_c of different PDDA_H:PPA–sand samples, together with the corresponding Re and C_d, are summarized in Table S1 and were used for subsequent shear-stress/force calculations.

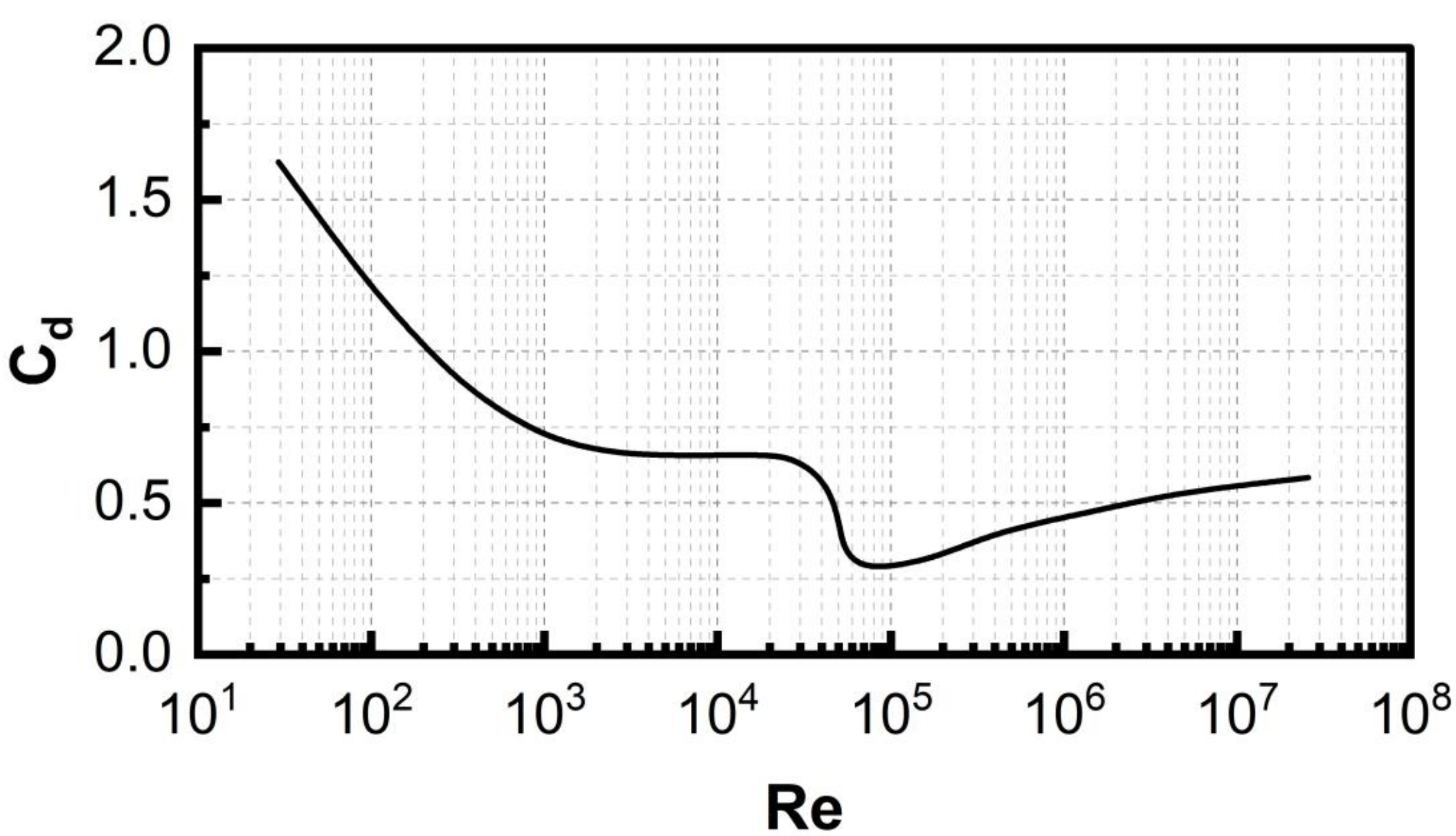

Figure S2. Drag coefficient C_d as a function of Reynolds number Re for a sphere in a Newtonian fluid.

Table S1. Critical rotational speed (n_c), Reynolds number (Re), and drag coefficient (C_d) for PDDA_H/PPA-sand clusters.

PDDA_H:PPA (molar ratio)	n_c (rpm)	Re	C_d
1:2	428.8	3.27×10^4	0.64
1:3	514.8	3.92×10^4	0.55
1:4	577.8	4.40×10^4	0.50
1:5	609.2	4.64×10^4	0.50
1:6	600.1	4.58×10^4	0.50
1:7	591.8	4.51×10^4	0.50
1:8	578.6	4.41×10^4	0.50

S4. Control experiments of single-polyelectrolyte systems and PDDA-based PECs systems

According to the molar amounts shown in Figure S3, the corresponding masses of each component were calculated based on the monomer molecular weights of the respective polyelectrolytes, namely PDDA = 161.1 g·mol^{-1}, PPA = 80.0 g·mol^{-1}, PSS = 208.0 g·mol^{-1}, PAA = 74.0 g·mol^{-1}, and phosphoric acid (PA), the monomeric analogue of PPA, = 98.0 g·mol^{-1}. The required amounts of each reagent were then weighed and mixed to prepare the PECs. Subsequently, the obtained PECs were mixed with 1 g of sand pre-wetted with 2 mL of water, and the aggregation behavior of the sand particles was observed.

	Single polyelectrolyte	PDDA-based PECs systems	
		PDDA_H:PPA = 1:1<br>(2.0×10^{-4}:2.0×10^{-4} mol)	PDDA_H:PPA = 1:5<br>(2.0×10^{-4}:1.0×10^{-3} mol)
**PDDA_H + Sand**<br>(2.0×10^{-4} mol + 1g)<br>(PDDA_H-DPn ≈ 2,787)	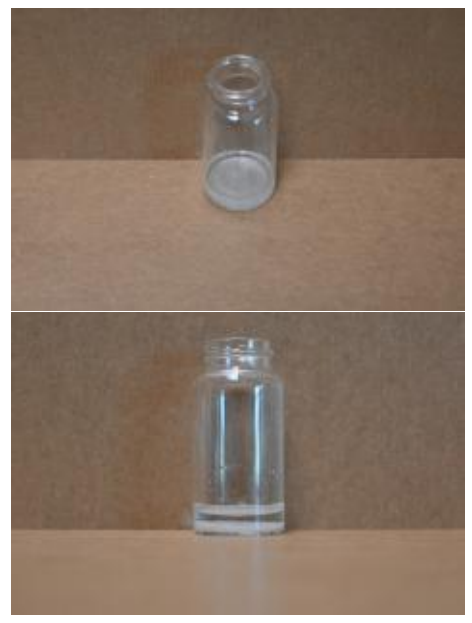		
**PPA + Sand**<br>(1.0×10^{-3} mol + 1g)<br>(PPA-DPn ≈ 6)	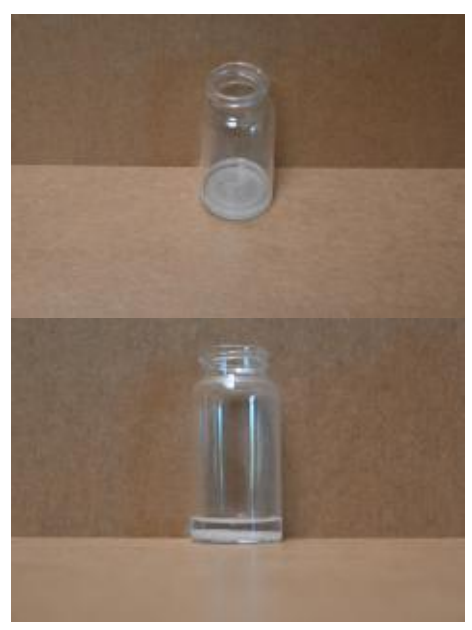		
**PAA+Sand**<br>(1.0×10^{-3} mol + 1g)<br>(PAA-DPn ≈ 16,891)	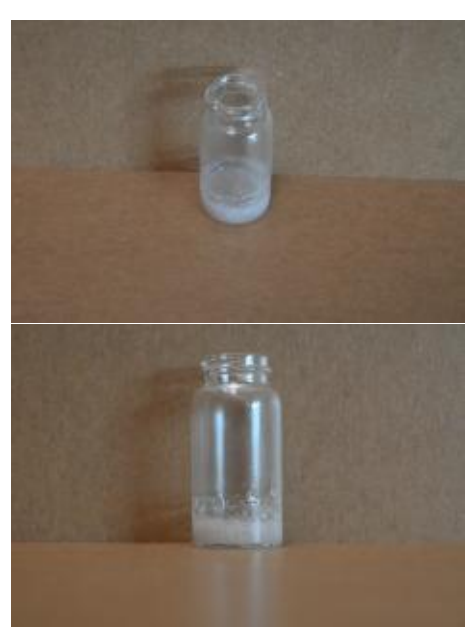		
**PSS + Sand**<br>(1.0×10^{-3} mol + 1g)<br>(PSS-DPn ≈ 343)	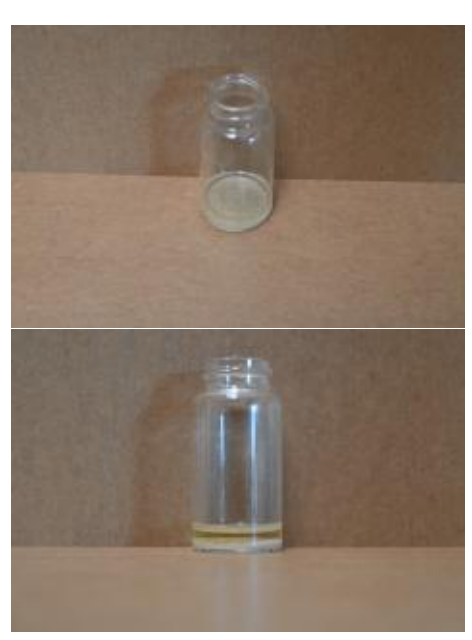		

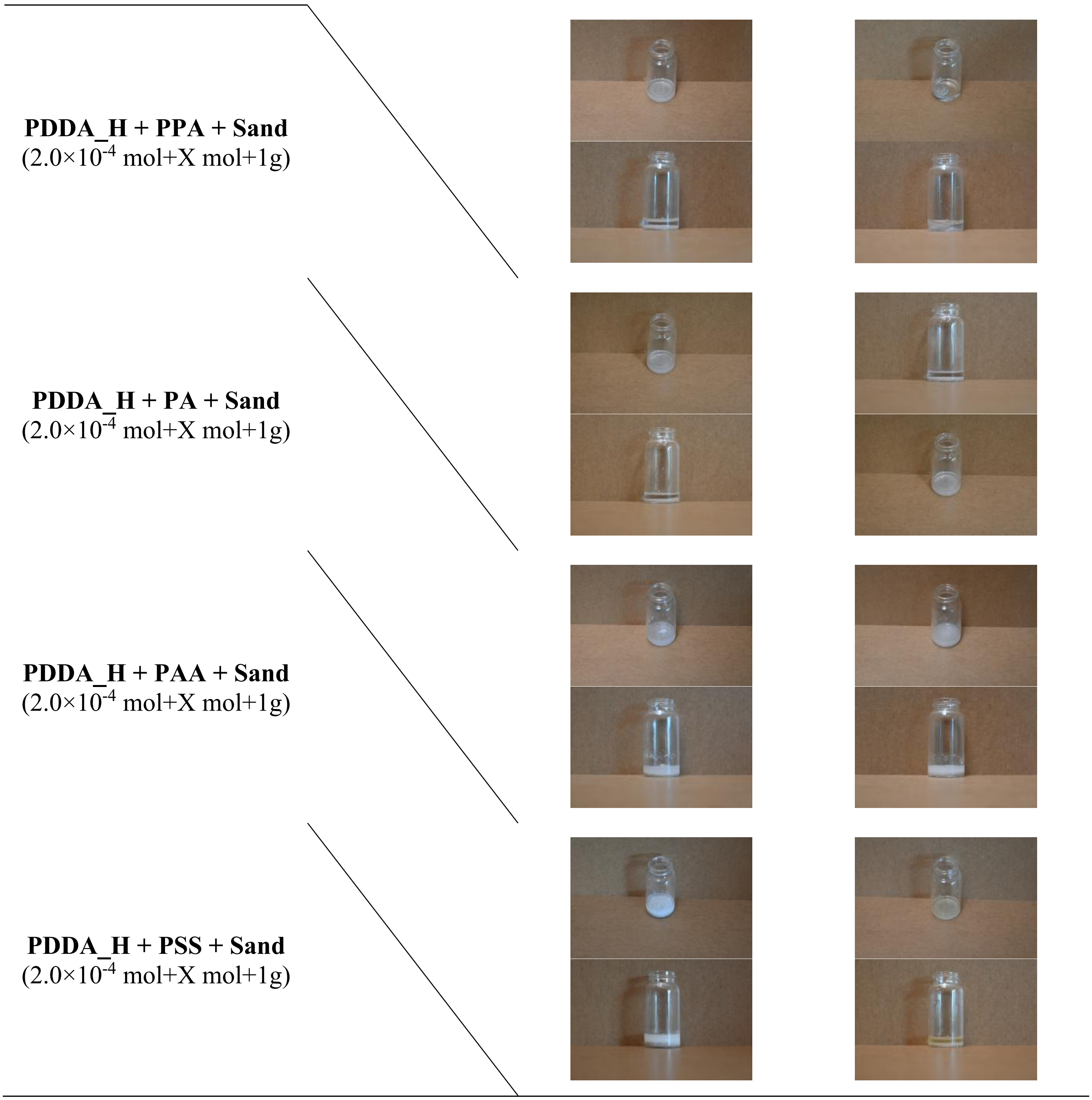

Figure S3. Comparison of sand-consolidation performance of single-polyelectrolyte systems and PDDA-based PECs systems with different polyanions.

As shown in Figure S3, in the single-polyelectrolyte systems, none of the samples showed obvious sand-consolidation performance. In the PDDA-based PECs systems, the combinations of PDDA_H with PSS or PAA showed no sand-consolidation effect and

formed white solid precipitates; this phase-separated state was also unsuitable for subsequent rheological testing. In contrast, only PDDA_H/PPA at an appropriate ratio, such as 1:5, exhibited clear sand-consolidation performance while remaining as a transparent liquid, making it suitable for rheological measurements. In addition, the absence of sand consolidation in the PDDA_H/PA system indicates the sand-stabilization performance of PDDA_H/PPA is not mainly caused by the pH decrease induced by PPA, but is more likely associated with the formation of a polyelectrolyte complex network between PDDA and polymeric PPA.

These control experiments indicate that the PDDA_H/PPA system has relatively better sand-consolidation performance and characterizability, making it a suitable system for further investigating the PECs network structure and its sand-stabilization mechanism.

S5. FT-IR Characterization of PDDA/PPA Polyelectrolyte Complexes

As shown in Figure S4, pristine PDDA showed a C–H stretching vibration band at 2929.0 cm^{-1} and a $–CH_2–$ bending vibration band at 1468.0 cm^{-1}. The band at 963.7 cm^{-1} was associated with the quaternary ammonium structure.[5] For pristine PPA, the band at 1201.6 cm^{-1} was assigned to P=O-related vibration, while the bands at 997.7 and 903.4 cm^{-1} were attributed to P–O and P–O–P related vibrations, respectively.[6, 7]

In the PDDA/PPA, some characteristic absorption bands showed obvious changes. For example, the band of PDDA at around 1468.0 cm^{-1} shifted to 1472.3 cm^{-1}. Meanwhile, the phosphate-related absorption bands of PPA at around 1201.6, 997.7, and 903.4 cm^{-1} shifted, overlapped, and mainly appeared as bands at around 1111.3 and 956.5 cm^{-1} in the

complex system. These changes indicate that the chemical environments of the functional groups changed after the complexation of PDDA and PPA, suggesting interactions between the two components. No obvious new characteristic absorption band was observed, suggesting that no new covalent chemical bonds identifiable by FT-IR were formed during the complexation process. Therefore, considering the positively charged quaternary ammonium groups in PDDA and the negatively charged phosphate groups in PPA, these results support that PDDA/PPA PECs were mainly formed through non-covalent interactions, in which electrostatic interactions may play an important role.

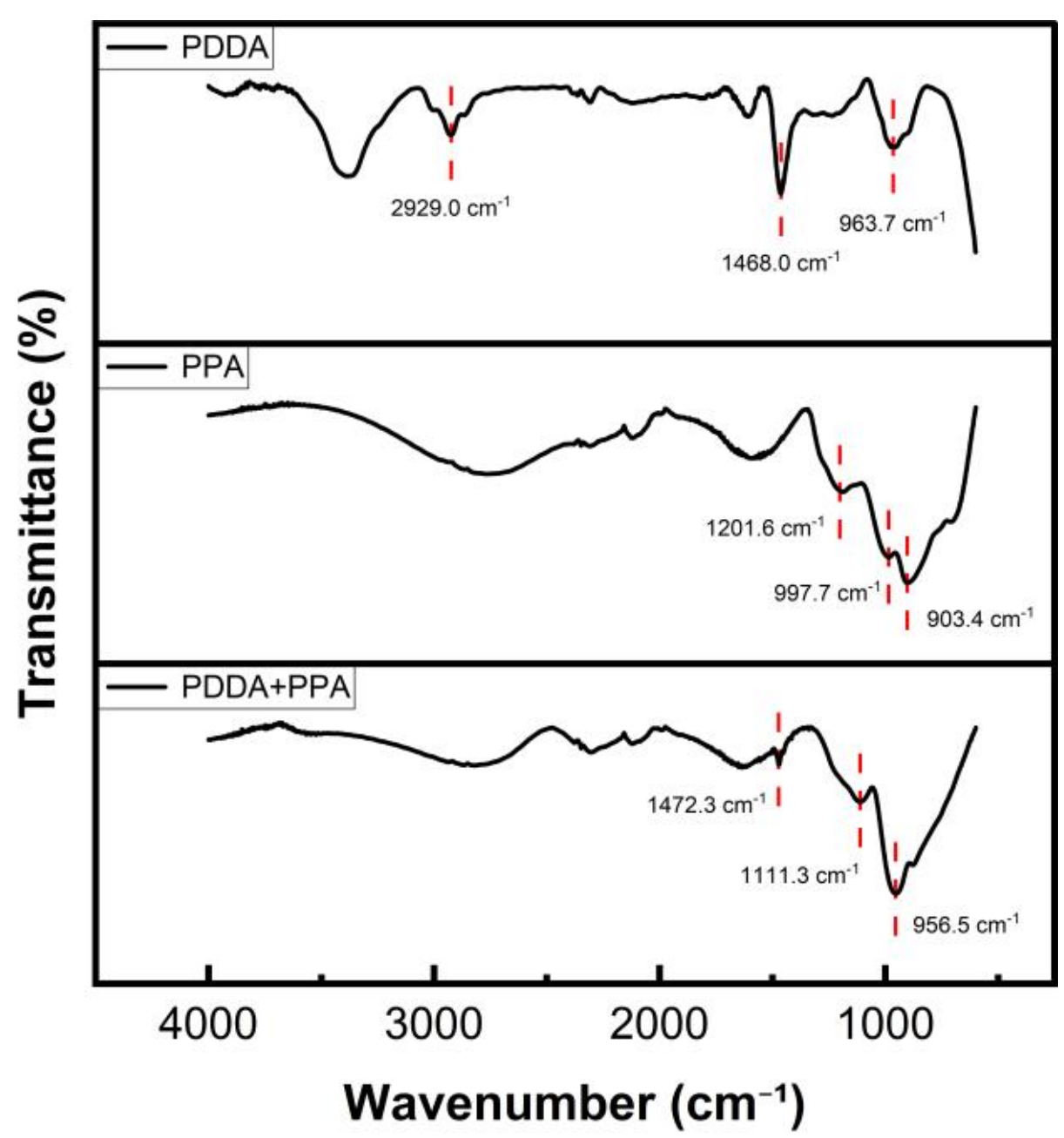

Figure S4. FT-IR spectra of PDDA, PPA, and PDDA/PPA.

S6. Multiple-Overtone QCM-D Responses during the Alternating Adsorption of PDDA_H and PPA on SiO_2 Surfaces

The multiple-overtone QCM-D data are shown in Figure S5. In the 3rd, 5th, and 7th overtones, the alternating addition of PDDA and PPA produced a clear stepwise adsorption response, indicating successive interfacial assembly of the PDDA/PPA layer on the SiO_2 surface.

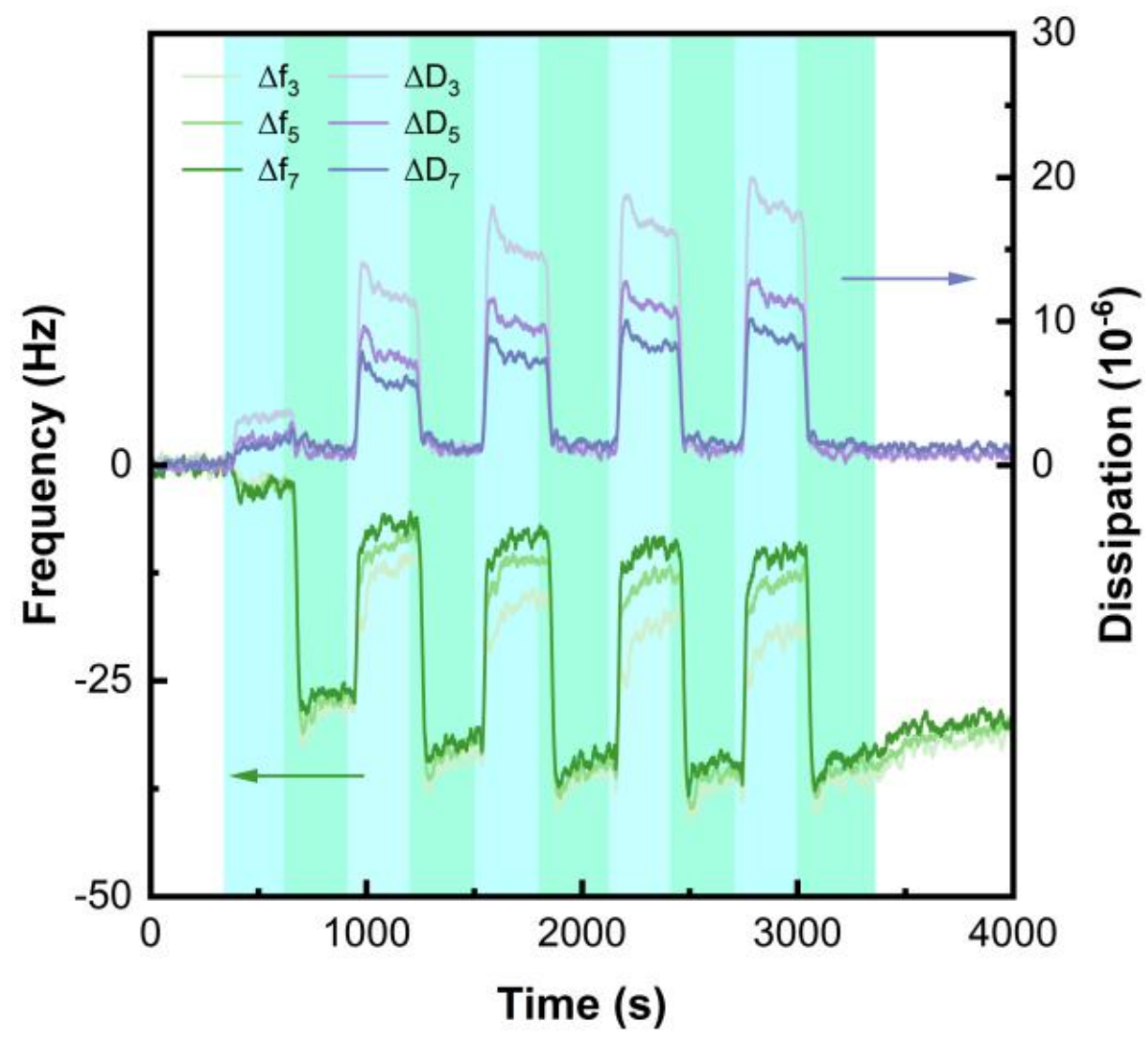

Figure S5. Real-time QCM-D responses for the stepwise assembly of PDDA_H/PPA on SiO_2 surfaces. Frequency shifts (Δf_3, Δf_5, and Δf_7, left axis) and dissipation changes (ΔD_3, ΔD_5, and ΔD_7, right axis) are shown. Light blue and light green regions indicate the injection periods of PDDA_H and PPA, respectively.

S7. Time-Resolved Photographic Snapshots of the Mechanical Stability Test (Extracted from Video Recordings)

During the mechanical stability test, the disintegration behavior of sand clusters in water under mechanical agitation was recorded as continuous video footage. Since video files cannot be directly included in the Supporting Information, the video was processed by extracting frames at a fixed time interval (every 1 s) to generate a time-resolved

sequence of photographic snapshots, which are compiled in Figure S6 to visually illustrate the evolution of cluster integrity with time.

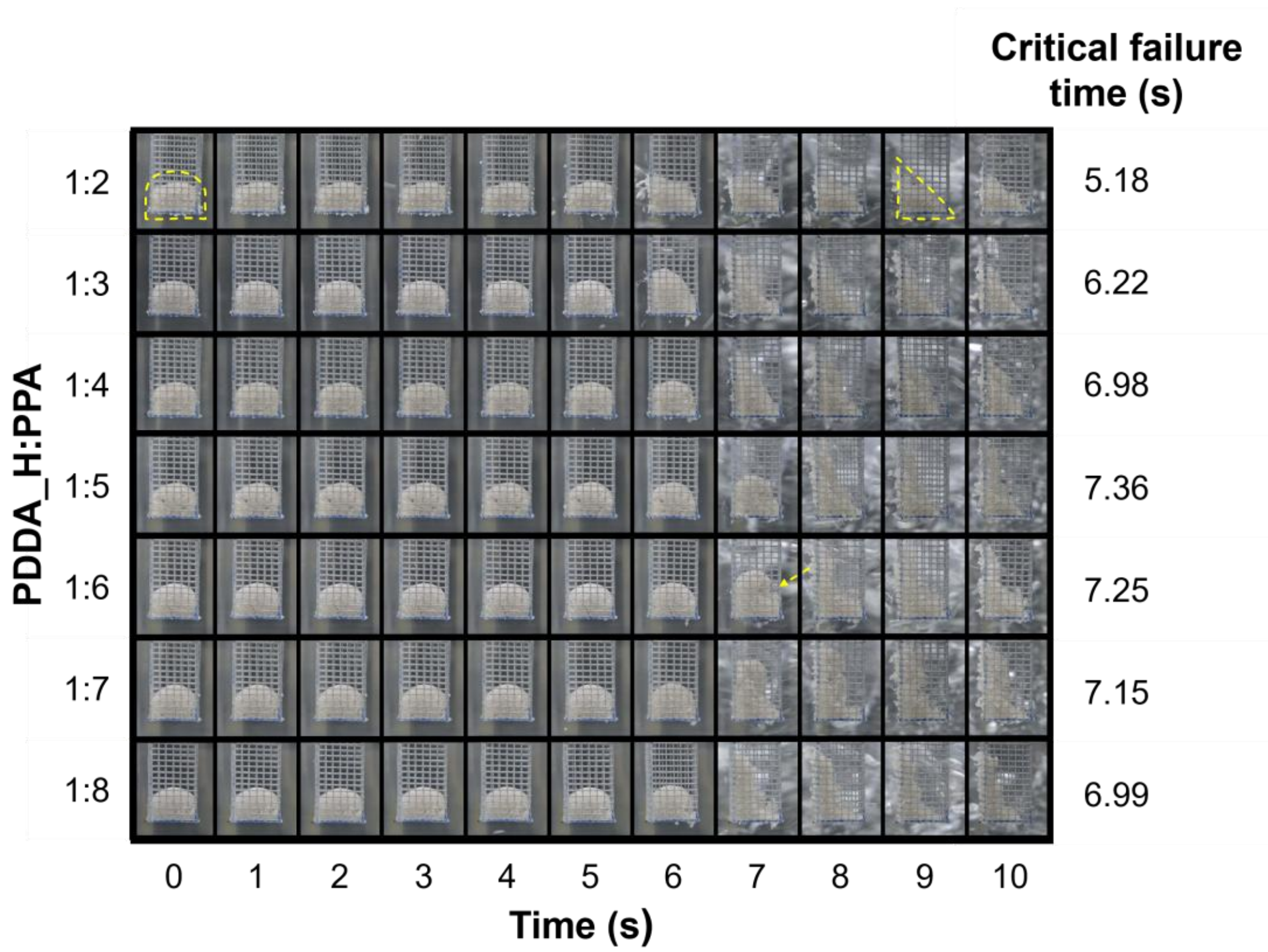

Figure S6. Time-resolved photographic snapshots showing the morphological evolution and critical disintegration of sand clusters under mechanical agitation.

During the test, the rotational speed was linearly increased from 0 to 660 rpm over a period of 0–9 s. Prior to water addition, the sample exhibited a nearly ideal spherical shape (see Figure 2c in the main text). After the sample was placed inside the mesh holder and immersed in water, its initial morphology deviated from a perfect sphere due to the combined effects of gravity, buoyancy, and mechanical confinement by the mesh, resulting in a confined quasi-spherical or hemispherical appearance (e.g., PDDA_H:PPA = 1:2 at 0 s). Despite this initial deviation, a clear and reproducible morphological evolution was

observed as the rotational speed increased. At the early stage, the cluster retained an overall quasi-spherical contour, followed by the onset of central cracking under stronger hydrodynamic forcing (e.g., PDDA_H:PPA = 1:6 at 7 s). With further enhancement of shear, the cluster progressively transformed into a pronounced triangular morphology (e.g., PDDA_H:PPA = 1:2 at 8 s), and subsequently underwent continued erosion and fragmentation under hydrodynamic shear until the end of the test.

In this study, the time point at which the sand cluster first transforms into a triangular morphology is defined as the critical disintegration time (the critical disintegration times for different PECs compositions are indicated on the right side of Figure S6). This time point is regarded as the transition from an overall mechanically stable structure to irreversible structural disintegration. Following the procedure described in the main text, the critical disintegration time is further converted into the corresponding critical rotational speed, and subsequently into the critical shear rate and critical shear stress, which are used to quantitatively evaluate the mechanical stability of sand clusters formed with different PDDA_H/PPA compositions.

REFERENCES

(1) Rumble, J. R. *CRC Handbook of Chemistry and Physics*; CRC Press: Boca Raton, FL, 2025.

(2) Mohr, P. J.; Newell, D. B.; Taylor, B. N. CODATA recommended values of the fundamental physical constants: 2014. *Rev. Mod. Phys.* **2016**, *88* (3). DOI: 10.1103/RevModPhys.88.035009.

(3) Hales, T. C. A proof of the Kepler conjecture. *Ann. Math.* **2005**, *162* (3), 1065-1185. DOI: 10.4007/annals.2005.162.1065.

(4) Achenbach, E. The effects of surface roughness and tunnel blockage on the flow past spheres. *J. Fluid Mech.* **1974**, *65* (1), 113-125. DOI: 10.1017/S0022112074001285.

(5) Mwangi, I. W.; Ngila, J. C.; Ndungu, P. A new spectrophotometric method for determination of residual polydiallyldimethylammonium chloride flocculant in treated water based on a diazotization-coupled ion pair. *Water SA* **2012**, *38* (5). 707-714. DOI: 10.4314/wsa.v38i5.8.

(6) Jastrzebski, W.; Sitarz, M.; Rokita, M.; Bulat, K. Infrared spectroscopy of different phosphates structures. *Spectrochim Acta A Mol Biomol Spectrosc* **2011**, *79* (4), 722-727. DOI: 10.1016/j.saa.2010.08.044.

(7) Popov, E. M.; Kabachnik, M. I.; Mayants, L. S. Vibrational Spectra of Organophosphorus Compounds. *Russ. Chem. Rev.* **1961**, *30* (7), 362-377. DOI: 10.1070/RC1961v030n07ABEH002989.
